# Investigating the Foraging, Guarding and Drifting Behaviors of Commercial *Bombus terrestris*

**DOI:** 10.1007/s10905-021-09790-0

**Published:** 2022-01-18

**Authors:** Ellen L MacKenzie, Dave Goulson, Ellen L Rotheray

**Affiliations:** School of Life Sciences, University of Sussex, Falmer, BN1 9QG UK

**Keywords:** Bumble bee, Specialization, Behavioral plasticity, Foraging strategy, Robbing

## Abstract

Social insects have high levels of cooperation and division of labor. In bumble bees this is partly size-based, with larger bees performing tasks outside the nest and smaller bees remaining inside, although bumble bees still display considerable behavioral plasticity. The level of specialization in tasks outside the colony, including foraging, guarding and drifting (entering a foreign colony), is currently unknown for bumble bees. This study aimed to assess division of labor between outside tasks and the degree of specialization in foraging, guarding, and switching colonies in commercially reared bumble bees placed in the field. Nine factory-bought *Bombus terrestris* colonies were placed on three farms in Sussex, UK, between June and August 2015. Forty workers from each colony were radio-tagged and a reader on the colony entrance recorded the date, time and bee ID as they passed. The length and frequency of foraging trips and guarding behavior were calculated, and drifting recorded. The mean (±SD) length of foraging trips was 45 ± 36 min, and the mean number of foraging trips per day was 7.75 ± 7.71. Low levels of specialization in guarding or foraging behavior were found; however, some bees appeared to guard more frequently than others, and twenty bees were categorized as guards. Five bees appeared to exhibit repeated “stealing” behavior, which may have been a specialist task. The division of labor between tasks was not size-based. It is concluded that commercial bumble bees are flexible in performing outside nest tasks and may have diverse foraging strategies including intra-specific nest robbing.

**Supplementary Information:**

The online version contains supplementary material available at 10.1007/s10905-021-09790-0.

## Introduction

Social insect societies are characterized by a high level of cooperation, with workers forsaking their own reproduction for that of the queen, and division of labor, where different individuals take on different tasks (Westhus et al. 2013). Task specialization can increase performance over time, thus contributing to increased efficiency (O’Donnell and Jeanne 1992; Oster and Wilson 1978), and the division of labor displayed in colonies has been credited with the ecological success of social insects (Wilson 1990; Goulson 2009).

Whilst honey bees (*Apis* sp.) display clear age-based division of labor (Robinson et al. 1989) and many ant species have morphologically distinct task forces (Powell and Franks 2006), behavioral specialization in social bumble bees (*Bombus* sp.) is less defined (Goulson 2009). There is some age-based division of labor in bumble bees, as recently emerged workers remain within the nest performing nursing tasks and will switch to foraging tasks later in their life (Cameron 1989; O’Donnell et al. 2000; Silva-Matos and Garófalo 2000). However, aside from very young bees, most tasks are performed by bees of any age (Cameron 1989), workers will switch from within-nest tasks to foraging if the conditions require it (Cartar 1992) and the age at which a bee will begin foraging is flexible (O’Donnell et al. 2000).

Bumble bees also display some size-based division of labor. Bumble bee workers exhibit extreme variation in size (Goulson et al. 2002) and larger bees tend to specialize in foraging whereas smaller bees usually remain within the nest (Free 1955; Goulson et al. 2002; Jandt and Dornhaus 2009; Yerushalmi et al. 2006). There is some evidence that larger bees are more efficient foragers, a potential explanation for the size-based division of labor (Goulson et al. 2002; Klein et al. 2017; Spaethe and Weidenmüller 2002). However, this was only the case for nectar foraging, and not pollen collection (Goulson et al. 2002; Spaethe and Weidenmüller 2002). There is considerable variation in size within foragers (Goulson et al. 2002), and it has been suggested that this may benefit the colony as foragers of different sizes tend to visit, and are morphologically suited to, different flowers (Peat et al. 2005). There does not appear to be a finer-scale division of labor of within-nest tasks, and bees of all sizes perform multiple tasks inside the nest (Jandt et al. 2009). Bumble bees, then, display considerable plasticity in their behavior (O’Donnell et al. 2000).

Although there is some understanding of the division of labor between within-nest and out-of-nest tasks, as well as between within-nest tasks, there is currently no research on the level of specialization among out-of-nest behaviors including foraging, drifting and guarding. Alongside foraging, out-of-nest tasks includes guarding behavior, where a bee will monitor the colony entrance and assess any incoming bees (Free 1958). Whilst guarding in honey bees is, to some extent, a distinct and specialist behavior (Moore et al. 1987), bumble bees do not perform particularly strict or efficient guarding, since they often allow non-nest mates to enter (Blacher et al. 2013a), and it is only exhibited in larger colonies (Free 1958). There is no evidence of age-related differences in guarding bumble bees (Cameron 1989; Free 1958), but, alongside foragers, guards are larger than bees that remain within the nest (Jandt and Dornhaus 2009). Bumble bees therefore appear to display flexibility in this behavior. There has, however, been no further research into the extent that bumble bees specialize in either guarding or foraging.

Another behavior displayed by bees performing out-of-nest tasks is drifting, where a bee enters a colony that is not its own (Birmingham et al. 2004). This behavior is common and has been observed in bumble bees (Zanette et al. 2014), honey bees (Chapman et al. 2009), stingless bees (Stephens et al. 2017), sweat bees (Soro et al. 2009) and wasps (Sumner et al. 2007). Although drifting may be enhanced in artificial settings with several colonies close to each other (Birmingham et al. 2004), it has also been observed in wild bumble bees (O’Connor et al. 2013; Takahashi et al. 2010), suggesting that this behavior occurs naturally in bumble bees (Zanette et al. 2014). Visits by drifters can be brief, but in some cases the bee will remain in the host colony and perform both within-nest tasks and foraging for its new colony (Lopez-Vaamonde et al. 2004; Pfeiffer and Crailsheim 1999). This is common in *Bombus terrestris* and up to a third of workers in a colony can be non-natal drifters (Lefebvre and Pierre 2007).

Drifting can occur accidentally through orientation errors (Chapman et al. 2009; O’Connor et al. 2013), but evidence suggests that in some cases workers seek out other colonies in order to lay their own male eggs, acting as reproductive parasites (reviewed in Beekman and Oldroyd 2008). Another form of directed drifting in bees is the stealing of food from nearby colonies. This intra-specific nest robbing has been reported in honey bees where it is more common when nectar resources are scarce (Downs and Ratnieks 2000). This can present a considerable threat for honey bee colonies, leading to colony death (Winston 1987), and so guarding behavior will increase when this threat is high (Downs and Ratnieks 2000). To our knowledge there are no existing data on nest-robbing in bumble bees; however, there are anecdotal references indicating that it does occur, albeit rarely (Blacher et al. 2013a; Free and Butler 1959).

While it has been observed that drifter honey bees carry out less foraging activity than their host nest-mates (Pfeiffer and Crailsheim 1999) and non-natal bumble bees perform different levels of brood care (Yagound et al. 2012), there has been little investigation into how stealing bumble bees and other drifters perform other tasks and fit into the division of labor system for out-of-nest behaviors.

Understanding division of labor is key to our knowledge of the ecological success of social insects (Wilson 1990). As the efficient allocation of labor is vital to a colony’s food intake (Kapustjanskij et al. 2007), division of labor and task specialization can give insight into how bees respond to changing food supplies, an increasing conservation concern (Blake et al. 2011). Drifting behavior also has conservation implications, as it can contribute to the spread of diseases (Fries and Camazine 2001).

This study aimed to use an automated data collection method to investigate the division of labor and specialization between tasks external to the nest - foraging, guarding and drifting - in captive-reared *B. terrestris* colonies. In particular, we asked: Do foraging, guarding and drifting behaviors differ for bees of different sizes and ages?; Do workers specialize in either foraging or guarding behavior?; Do drifters perform foraging and guarding tasks differently? As the bees used in this study were commercially reared, the measured behaviors may differ from that of wild bees due to possible differences in foraging motivation or cognitive abilities. This study is therefore specifically investigating these behaviors in commercial bees, and demonstrates a potential methodology for collecting data on other bee populations.

## Materials and Methods

### Experimental Set-up

Nine Biobest *B. terrestris* colonies, each with a queen, were purchased from Agralan Ltd., UK. Thirty worker bees from each colony were captured in a red-lit room using tweezers and were transferred into individual tubes. The workers were then placed into queen marking tubes in the lab and a radio-frequency identification (RFID) tag was attached to the thorax using glue. The tags and readers were a standalone MAJA system from Microsensys (see Heidinger et al. 2014). A thorax width measurement was taken for each bee tagged in the lab. The bees were then placed back into their colony. A further 10 workers per colony were captured and tagged in the field after two weeks, however no thorax measurement was taken. In total, 360 bees were tagged.

Three colonies were each placed in Saddlescombe farm (50.889°N, −0.192°W), Plumpton farm (50.908°N, −0.068°W) and Gipps farm (50.954°N, 0.038°E) in Sussex, UK. Colonies were placed under shelter and were positioned at least one meter apart from each other with the entrances oriented in different directions by at least 90° to limit confusion between colonies. All colonies were placed in the field by the 29th of June 2015. Data from tagged bees were obtained from the Gipps colonies between 4th June and 3rd August 2015, Saddlescombe colonies between 16th June and 28th July, and Plumpton between 18th June and 19th July. Each colony had one RFID reader at the entrance which recorded the date, time and unique identifier (UID) of tagged bees as they entered and exited the colony. The data were then downloaded from the reader onto a computer.

### Data Collection

The raw data downloaded from the reader included the reader ID, bumble bee UID, the date and the time at which the bee passed the reader. Data was collected from a total of 89 bees from Gipps, 101 from Saddlescombe and 19 from Plumpton: 209 in total. Fewer records were obtained from Plumpton as one of the colonies failed. Records were only obtained from 209 of the 360 tagged bees, which could be a result of tags getting lost, faulty tags, or tagged bees remaining inside the colony for the duration of the data collection. The number of readings per bee varied from one to 2768. The longevity of each bee was defined as its lifetime within the study, calculated as the total number of days from the date of the first reading to the last reading inclusive. For each bee, the time interval between each reading was calculated and these intervals were categorized into different behaviors: foraging, guarding, stealing, visiting or switching. Foraging was defined when a bee was recorded repeatedly exiting for ≥10 min and re-entering the colony for a short time interval (1–5 min); guarding behavior was defined when a series of repeated one-minute records were recorded for at least 5 consecutive minutes, suggesting that the bee was remaining at the entrance of the colony for a period of time and repeatedly triggering the reader; “stealing” (so not to be confused with nectar robbing in bumble bees) was defined when a bee was recorded entering a neighboring colony and re-entering its own colony more than once, and immediately repeating this behavior; visiting was defined when a bee entered a different colony but did not repeat the behavior; bees that permanently entered a neighboring colony were classed as “switching” (see Online Resource 1 for more detail and caveats regarding these definitions).

### Statistical Analysis

All statistical analyses were carried out in R v4.0.1 (R Core Team 2020) (see Online Resource 4 for code). The data were analyzed using general linear models (GLMs), general linear mixed models (GLMMs) and binomial generalized linear models in the package lme4 (Bates et al. 2015). The final models are detailed in Table 2 in Online Resource 2.

For each model, the correlations between explanatory variables were assessed using Spearman’s rank correlation coefficient and only one of any highly correlating variables was included. In the mixed models the variable *colony* was included as a random intercept to account for repeated measures, except in models where the data came from fewer than 5 colonies, as variables with few levels are best included as fixed effects. For this reason, *site* (site 1: Gipps, 2: Saddlescombe and 3: Plumpton) was also included as a fixed effect despite the repeated measures design. To assess the effect of explanatory variables on binary response variables (occurrence of guarding, stealing and switching), generalized linear models were produced with the binomial error family and the complementary log-log link function. This link function does not assume a symmetrical distribution of 1 and 0 values (Chen et al. 1999). When the inclusion of *colony* as a random variable in the binomial models was too complex for the data, it was instead included as a fixed effect.

Model selection was conducted by comparing corrected Akaike Information Criterion (AICc) values of candidate models and the model with the lowest AICc was selected. Model assumptions were checked using diagnostic plots and, where necessary, the response variable was square root or log transformed.

The significance of the effect of explanatory variables in the mixed models was obtained using the Kenward-Roger approximation in package lmerTest (Kuznetsova et al. 2017). This is acknowledged to be an effective and conservative method for the estimation of p-values and produces fewer type 1 errors than alternative methods (Halekoh and Højsgaard 2014; Luke 2017; Kuznetsova et al. 2017).

The variables used to assess foraging behavior were the mean length of foraging trip (hours) and the mean number of foraging trips per day for each bee. The relationship between these variables and longevity and thorax width were assessed using GLMMs. The change in foraging behavior over the bees’ lifetime was also assessed using GLMs where *bee* was included as a random effect. The variables used to assess guarding behavior were the occurrence of guarding (*guard*) and the mean length of guarding activity (hours). GLMs, GLMMs and a binomial generalized linear model were used to assess the relationship between guarding behavior and longevity, thorax width and foraging behavior. The variable used to assess stealing activity was the occurrence of stealing (*stole*). GLMs, GLMMs and binomial generalized linear models were used to assess the relationship between stealing and longevity, thorax width and foraging behavior. Models were made for all bees and also for bees only from Gipps farm, to investigate the stealing behavior at this location. The variable used to assess switching behavior was the occurrence of switching (*switched*). A general linear mixed model and a binomial generalized linear mixed model were used to assess the relationship between switching and longevity, thorax width and foraging behavior.

## Results

### Foraging

Of the 209 tagged bees that were detected by the reader, 159 exhibited foraging behavior. Those that did not forage had very few readings and so either died near the beginning, lost their tag or remained within the colony for the duration of the study. The mean (±SD) foraging trip length was 45 ± 36 min and the mean number of foraging trips per day was 7.75 ± 7.71. The mean thorax width of foragers was 5.42 ± 0.39 mm. Forager longevity ranged from 1 to 48 days with a mean of 11.99 ± 10.40 days.

The foraging models revealed that there was no significant difference in mean foraging time or foraging trips per day between bees of different thorax size (model 3: *p = 0.551*, model 4: *p = 0.246*. See Online Resource 2 for table of coefficients for each model). Similarly, neither foraging variable significantly influenced the longevity of the bees (model 1: *p = 0.076* and model 2: *p = 0.322*).

When foraging behavior over a bee’s lifetime was investigated, there was no change in the mean foraging trip length or number of trips per day over time (model 5: *p = 0.535*, model 6: *p = 0.380*).

### Guarding

Twenty individuals, from two Gipps colonies and two Saddlescombe colonies, displayed guarding behavior. The number of times these bees guarded ranged from one to 30. The length of time spent guarding ranged from six minutes to eight hours and 20 min. The mean (±SD) length of time spent guarding was one hour 37 min ±4 h 52 min.

**Fig. 1:**
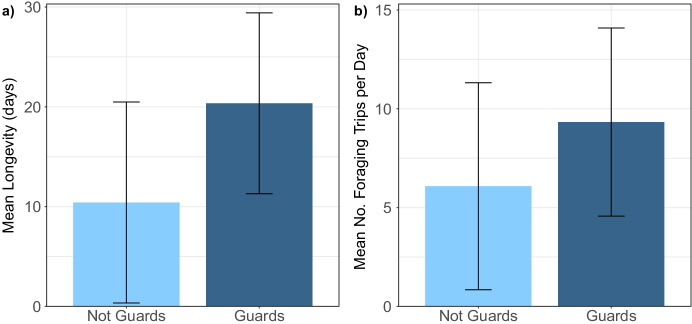
a) The mean (±SD) longevity (days observed within the study) for bees that did not guard and bees that guarded, *n* = 173. b) The mean (±SD) number of foraging trips per day for non-guards and guards, *n* = 173

Guarding bees were found to be larger and live longer than tagged bees that did not guard; however, they did not display a significant difference in foraging behavior. Guards had a mean thorax width of 5.35 ± 0.29 mm. This was not significantly different from the thorax width of non-guards, who had a mean of 5.45 ± 0.39 mm (model 13: *p = 0.384*, Supplementary Fig. 1a in Online Resource 3). Thorax width also had no significant influence on the mean length of time a bee spent guarding (model 10: *p = 0.129*). Guards lived between 6 and 41 days with a mean longevity of 20.35 ± 9.06 days. This was significantly higher than tagged bees that did not guard, who lived for 10.41 ± 10.07 days on average (model 7: *p = 0.003*; Fig. 1a).

Guards were not found to have a significantly different number of foraging trips per day compared to bees that did not guard (model 9: *p = 0.382*; Fig. 1b). There was also a non-significant difference in the mean length of foraging trips for guarding bees (model 8: *p = 0.469*; Supplementary Fig. 1b in Online Resource 3). There was also no significant effect of mean guarding time on either foraging variable (model 11: *p = 0.498* and model 12: *p = 0.660*).

### Stealing

Overall, 26 individuals, 7.2% of all tagged bees, entered a foreign colony during the study. Five bees exhibited stealing behavior (1.4%) as defined in the methods section, and in more detail in Online Resource 1. Only bees from Gipps farm showed stealing behavior. These bees came from all three Gipps colonies and stole from two colonies; one was robbed 30 times and the other six times. Three of the five bees had also switched colony earlier and were thus stealing from their original colony. The number of stealing events per bee ranged from two to 26 (Table 1). The highest number of stealing events by an individual in a single day was 10. Eleven further bees also switched colony, but did not exhibit stealing behavior. A further 10 bees were classed as “visitors”, where they entered a different colony but were not considered stealing or switching as they did not enter this colony multiple times within one day, nor did they stay permanently.

**Table 1:** The total number of times each “stealing” bee entered a foreign colony, the number of times each bee was considered to be stealing, the number of foreign colonies each bee entered, the number of colonies each bee was considered to have stolen from and the number of days where the bee displayed stealing behavior

“Stealing” Bee	No. times entered a foreign colony	No. stealing events	No. foreign colonies entered	No. colonies stole from	No. days stole on
**A**	7	2	1	1	1
**B**	2	2	1	1	1
**C**	7	4	2	1	1
**D**	10	5	1	1	2
**E**	35	26	1	1	7

Bees that stole had a mean (±SD) thorax width of 5.97 ± 0.18 mm, compared to a mean thorax width of 5.44 ± 0.38 mm for all measured bees that did not exhibit stealing behavior. This was not a statistically significant difference in size when assessed for all bees, nor for bees from just Gipps farm, where all stealing events occurred (model 20: *p = 0.092*, model 21: *p = 0.092*; Supplementary Fig. 2a in Online Resource 3). Stealing bees lived between 7 and 31 days with a mean longevity of 14.60 ± 9.96 days. This was not significantly different to the longevity of all non-stealing bees (model 14: *p = 0.910*), nor to the longevity of non-stealing bees from Gipps (model 17: *p = 0.720*; Supplementary Fig. 2b in Online Resource 3).

**Fig. 2:**
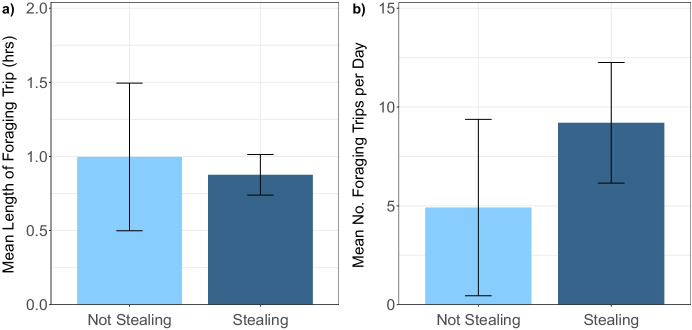
a) The mean (±SD) length of foraging trip for bees at Gipps farm that were considered to be stealing and bees that did not steal, *n* = 62. b) The mean (±SD) number of foraging trips per day for non-stealing and stealing bees at Gipps, *n* = 73

When all bees are considered, the occurrence of stealing did not significantly influence the mean foraging time or the number of foraging trips per day (model 15: *p = 0.839* and model 16: *p = 0.103*). When modelled with only bees at Gipps, stealing bees did not significantly differ in the length of foraging trip on average (model 18: *p = 0.768*; Fig. 2a); however, stealing bees at Gipps had a significantly higher number of foraging trips per day compared to other bees at Gipps (model 19: *p = 0.047*; Fig. 2b). No bees were found to both steal and perform guarding behavior.

### Switching

In total, 14 bees permanently switched to a different colony and carried out foraging activity at their new colony (3.8% of tagged bees). Three of these also displayed guarding behavior and three stole from another colony. Eight bees switched within the first day they emerged from the colony, and three of these switched after spending several hours outside of the colony. Switching bees had a mean (±SD) thorax width of 5.46 ± 0.51 mm, compared to 5.44 ± 0.37 mm for non-switchers. There was therefore no significant size difference between bees that switch colony and bees that do not (model 25: *p = 0.570*; Supplementary Fig. 3a in Online Resource 3). Switching bees lived for between 5 and 34 days, 14.71 ± 8.6 days on average, compared to a mean longevity of 11.28 ± 10.53 days for bees that did not switch. There was no significant difference between the longevity of switching and non-switching bees (model 22: *p = 0.142*; Supplementary Fig. 3b in Online Resource 3).

Bees that switched did not have a significantly different mean foraging time or mean number of foraging trips per day compared to bees that remained in their original colony (model 23: *p = 0.773*; Supplementary Fig. 3c in Online Resource 3, model 24: *p = 0.322*; Supplementary Fig. 3d in Online Resource 3).

## Discussion

### Foraging

This study aimed to assess the influence of worker size on the division of labor of foraging, guarding and drifting, the impact of these behaviors on longevity and the extent to which they are specialized tasks within commercial bumble bee colonies. Contrary to our predictions, no relationship between body size and foraging trip length or number per day was found, indicating a lack of sized-based division of labor among foragers. This suggests that bees of different sizes did not differ in the amount of foraging and so there was not size-based division of labor within foraging.

Previous studies on *B. terrestris* also found no difference in foraging time or number of trips between small and large bees (Spaethe and Weidenmüller 2002). Larger bees can carry larger loads (Fisher 1987; Goulson et al. 2002), and so by collecting their loads in the same time as smaller individuals, large bees are being more efficient. This is a possible adaptive explanation for the size difference between foragers and house-bees (Goulson 2009) and may indicate that these bees do, in fact, perform foraging behavior differently depending on size. However, this size-based difference in efficiency is only apparent in nectar foraging, not pollen foraging (Goulson et al. 2002; Spaethe and Weidenmüller 2002), and without measuring forage loads directly, true efficiency cannot be determined here. Other aspects of foraging can be influenced by size, including handling time, choice of flowers (Stout 2000; Peat et al. 2005), and response to predators (Gavini et al. 2020). Therefore, the division of labor within foraging tasks may be more complex than indicated by this study, and it may also be that the behavior of wild bees differs from our captive-bred bumble bee population. However, as bumble bees have flexible foraging strategies, any further specialization within foraging may be weak or temporally variable (Leonhardt and Blüthgen 2012; Russell et al. 2017).

The results also indicated that foraging activity did not significantly influence the mean longevity. This is surprising as foraging is risky and usually results in mortality (Cameron 1989; Jandt and Dornhaus 2011), and so it might be expected that higher foraging activity would reduce longevity. However, guard bees were found to live longer than non-guarding foragers and so the risk of foraging behavior to longevity cannot altogether be ruled out. A further surprising result was that foraging activity did not significantly decrease throughout a bee’s lifetime. This is in contrast to previous work which observed bumble bees foraging less as they age, eventually “retiring” to within-nest tasks (Cameron 1989). Workers might still have switched to within-nest tasks later in their life, as the final reading did not necessarily indicate a bee’s death, and it could have remained within the nest instead. The level of foraging activity is also dependent on the needs and size of the colony, which could influence the amount of foraging carried out each day (Weinberg and Plowright 2006). Furthermore, the artificial nature of the colony placement and the composition of commercially-reared bee colonies could also alter the foraging behavior of the colonies.

### Guarding

Twenty bees were classed as guards and these bees did not significantly differ in thorax width compared to tagged bees that did not guard. This suggests that there is not size-based division of labor between guarding and foraging in these colonies. All guards also foraged and did not significantly differ in their foraging activity. Foraging was also not significantly influenced by the average length of time spent guarding. These results indicate that guards did not perform less foraging and that foraging did not decrease with increased guarding. This suggests minimal specialization in guarding as opposed to foraging, as predicted.

There may, however, have been specialization to some extent. Although non-significant, guards made more foraging trips per day on average, as well as performing guarding tasks. This implies that the guards were busier than non-guards and carried out more work. Guard honey bees are very active (Moore et al. 1987), and bumble bee activity level may influence spatial distribution within the nest and thus the tasks undertaken (Jandt and Dornhaus 2009). So, division of labor of outdoor tasks in this case may be based on activity level, with “hard-working” bees guarding. This is contradicted, however, by the fact that the guards had a significantly higher longevity compared to foraging bees that did not guard. This is surprising, as higher risk activities such as foraging and guarding can decrease longevity in bees (Jandt and Dornhaus 2011; O’Donnell and Jeanne 1995), as can high energy expenditure and a higher workload (Kelemen et al. 2019; Schmid-Hempel and Wolf 1988). This may have been influenced by the foraging or guarding needs of these particular colonies, and the placement of the colonies could have affected risk and therefore mortality.

There is further evidence that these bees specialize in guarding to some extent, as few tagged bees guarded and seven did so more than once, in one case 30 times. Guarding activity did not appear to be widely shared, with many bees guarding a few times, but rather fewer bees specialized in performing it more often. This was found to be similar in a study on honey bees, where bees that guard more than once are more likely to do so again (Moore et al. 1987). For both honey bees and these tagged bumble bees however, most individuals did not guard for long and, in the present study, most only guarded once. This could suggest that learning and experience are not required for guarding and so specialization is limited (Moore et al. 1987), which may apply to these bumble bees. Honey bees appear to exhibit stronger specialization than the bumble bees in this study, as once they foraged they no longer guarded (Moore et al. 1987), whereas tagged bumble bees more frequently switched between these tasks. The behavioral flexibility observed in these bees is consistent with previous research on bumble bees which found some short-term but overall weak specialization in within-nest tasks (Jandt et al. 2009).

These results may indicate a lack of selection pressure for task specialization. Bumble bees show minimal guarding, and only in larger colonies (Free 1958). Bumble bee guards are also fairly tolerant of non-nestmates (Blacher et al. 2013a). This could be because the threat of intra-specific nest-robbing is low (Free and Butler 1959) and drifted bumble bees can benefit the host colony by working (Blacher et al. 2013a). Therefore, acceptance of non-nestmates and low investment in guarding may have been selected for in bumble bees (Blacher et al. 2013a; Downs and Ratnieks 2000).

### Drifting

Twenty-six bees drifted during the study, 7.2% of all bees tagged. Fourteen drifters stayed permanently in their host colony (3.8%) and five were classed as stealing (1.4%). These estimates are fairly low compared to other studies, which have observed up to 50% of workers drifting, however these rates are highly variable across studies and colonies (Birmingham and Winston 2004; Blacher et al. 2013b; Lefebvre and Pierre 2007). Colonies at each site were placed within two meters of each other, which may influence these results (Downs and Ratnieks 2000; Lopez-Vaamonde et al. 2004); however, it is accepted that drifting itself is not solely a result of artificial settings (O’Connor et al. 2013; Takahashi et al. 2010).

“Stealing” bees were not significantly different in size than bees that did not exhibit stealing behavior. Similarly, there was no observable size difference between bees that switched colonies and those that remained. As with guarding, drifting therefore did not appear to be a specialization of either small or large bees in this case, further suggesting that there was not strict size-based division of labor between out-of-nest activities. Whilst there have been no studies investigating or reporting stealing behavior in bumble bees, these data are consistent with previous work on *Bombus occidentalis* where drifters were reportedly no larger than resident bees (Birmingham et al. 2004).

Bees that switched colony did not have a significantly different longevity compared to bees that remained in their original colony. In addition, “stealing” bees did not significantly differ in their recorded longevity compared to bees that did not appear to steal. Lifespan of drifting bumble bees has not been previously investigated; however, honey bee drifters may have increased longevity, attributed to the fact that they are less active and do less foraging than resident bees (Dornhaus and Chittka 2005). In contrast, so-called “risky robbing” in honey bees, where honey is stolen from other honey bee colonies, has been associated with a shorter life span compared with foraging honey bees (Kuszewska and Woyciechowski 2014). The minimal impact of drifting on longevity in the present study may suggest that stealing was not necessarily a “risky” foraging strategy for these bumble bees. An assessment of the level of risk of stealing compared to other foraging strategies may be an interesting subject for further research.

To our knowledge, this is the first investigation into drifter bumble bees’ performance of foraging and guarding. Bees that appeared to steal also had more foraging trips per day on average than other Gipps bees that did not steal. Stealing bees therefore seemed to conduct more foraging trips of roughly the same time, suggesting that they were more active foragers. Switching bees did not have different foraging activity to bees that did not switch. This, alongside the fact that three switched bees were classed as guards, suggests that these bumble bees became highly integrated into their host colony’s workforce, unlike honey bees which were found to perform very little work for their host colony in a previous study (Pfeiffer and Crailsheim 1999).

Whilst it is known that bumble bees may seek out other colonies to lay their eggs in, acting as reproductive parasites (Birmingham et al. 2004), in this case the majority of switching bees switched within the first day or after they spent a long period outside the colony, suggesting orientation errors may be responsible for the switching behavior.

Surprisingly, three bees categorized as stealing had also switched colony and were considered to be stealing from their original colony. There has been no report of this behavior previously, aside from an observation of a honey bee commuting between its host and original colony (Neumann et al. 2001). For all five stealing bees, the stealing was highly directional, and no bee was considered to be stealing from more than one colony. Furthermore, the extent to which some bees stole indicates that this was not accidental drifting. One bee entered its target colony 35 times, 26 of which were categorized as stealing, and another entered 10 times, five of which were considered stealing. The directional and consistent drifting of these bees implies intent.

The data also suggests that the stealing observed in these bees may be a specialized behavior, as stealing was observed repeatedly in only a few bees. Free and Butler (1959) observed that a bumble bee may repeatedly steal food from other colonies, similar behavior to the bees in this study. Stealing may therefore be considered a foraging strategy employed by some bumble bees. The mechanism behind this specialization is still unclear and, as outlined above, is likely not size-based in this case. It would therefore be interesting to investigate further characteristics of stealing bees.

Stealing was only observed at one site: Gipps farm. This suggests that there may have been environmental factors determining stealing behavior. Honey robbing in honey bees is dependent on availability of forage and can cease when nectar availability is high (Downs and Ratnieks 2000). Since bumble bee foraging activity is also known to vary depending on food availability and colony conditions (Cartar 1992; Weinberg and Plowright 2006), it is possible that the stealing observed in these bumble bees may be a temporary strategy depending on current colony and environmental conditions.

Taken together, these drifting results suggest a level of behavioral flexibility in these colonies. This is consistent with previous research which has also highlights flexibility in bumble bees (see O’Donnell et al. 2000).

## Conclusion

This study provides insight into the specialization and division of labor of behaviors performed by commercial bumble bees outside of the nest: foraging, guarding and drifting, including stealing and switching colonies. As predicted, these bumble bees did not appear to exhibit strong specialization in these tasks, and some perform all three. There was no significant difference in size between bees performing different behaviors, suggesting that the size-based division of labor observed between house-bees and those carrying out external tasks does not further divide outdoor tasks. Whilst no bee solely performed guarding or stealing behavior, there did appear to be some level of specialization in these tasks with few bees performing them repeatedly.

This is the first study to quantify the frequency of stealing behavior in bumble bees. Bees that switch colony may be doing so accidentally; however, the highly directional and persistent nature of the drifting behavior recorded in this study suggests that these bees may be deliberately stealing food from their neighbors. We conclude that captive bumble bees display little specialization in guarding behavior, however, are capable of exhibiting directional and consistent intra-specific nest robbing, indicating plasticity in both outside of nest tasks and foraging strategies. Whilst these conclusions can only be applied to the commercially-reared bees in this study, due to potential behavioral differences compared to other bee populations, this study demonstrates the feasibility of using automated data collection as a means of investigating the daily behaviors of bees.

## Supplementary Information

ESM 1 (PDF 169 kb)

ESM 2 (PDF 228 kb)

ESM 3 (PDF 80 kb)

ESM 4 (PDF 213 kb)

## Data Availability

Data made available with submission.

## Code Availability

R software code is appended as Supplementary Information.
