# Supplementary material for: Investigating the Foraging, Guarding and Drifting Behaviors of Commercial Bombus terrestris
Source: J Insect Behav. 2022 Jan 18;34(5-6):334–45. doi: 10.1007/s10905-021-09790-0 (PMC8813815; doi:10.1007/s10905-021-09790-0)
Supplement: Supplementary file 1 — (PDF 169 kb) [file 10905_2021_9790_MOESM1_ESM.pdf]

Investigating the foraging, guarding and drifting behaviors of commercial *Bombus terrestris*

Journal of Insect Behavior

Ellen L MacKenzie[1], Dave Goulson[1] and Ellen L Rotheray[1]

Affiliations: [1] School of Life Sciences, University of Sussex, Falmer, BN1 9QG, UK

Corresponding Author: Ellen L MacKenzie, ellenmackenzie12@gmail.com

Supplementary Information 1: Behavior Classification

As there was only one RFID reader at each colony, and the bees could potentially be missed by the reader, it was not known if the bee was entering or exiting the colony at each reading. Therefore, behaviors were categorized based on the following assumptions.

It was initially assumed that a bee's first reading was it leaving the colony, and therefore the first time interval was spent outside the colony. The second reading was therefore considered to be the bee returning and the second time interval spent inside the colony, and so on. This initial pattern of "outside colony" then "inside colony" was repeated until a clear pattern of foraging behavior (outlined below) required it to be reversed. When the first time interval was longer than three hours this was classed as "inside colony" rather than outside as foraging flights did not tend to exceed three hours.

Foraging: When a bee displayed a repeated pattern of a longer time interval (≥10 minutes) followed by a short time interval (of only a few minutes), this was assumed to be foraging behavior with the longer time interval being the foraging flight. This assumption was based on the fact that foraging bumble bees repeatedly leave the colony for foraging flights of around an hour and will then return to the colony for a few minutes to deposit their forage before departing again (Dornhaus and Chittka 2005; Heinrich 1979). Flights were considered to be foraging when they lasted 10 minutes or longer, as orientation and defecation flights can last up to 10 minutes (Stelzer et al. 2010; Spaethe and Weidenmüller 2002) and bumble bees that do forage for less than 10 minutes do not return with a significant amount of forage (Spaethe and Weidenmüller 2002). If this foraging pattern was clear in a bee's first readings,

then this assumption overrode the initial assumption that the first reading was the bee leaving the colony.

Short Flight: When a bee exited the colony for less than 10 minutes this was categorized as a "short flight" and was not considered to be foraging behavior.

Inside 1 – Depositing Forage: If the time spent inside the colony between foraging flights was less than 15 minutes this was classed as "inside 1" behavior, where the bee is depositing its forage and/or resting between flights.

Inside 2 – Other Activities: When the time spent inside the colony lasted 15 minutes or longer this was considered "inside 2" behavior where the bee may have been undertaking other activities within the colony, resting for a longer period of time or remaining idle. This marked the end of a bout of foraging behavior. The limit of 15 minutes was chosen to distinguish between "inside 1" and "inside 2" behavior as time inside the colony was usually either very short, at five minutes or less, or was longer than 15 minutes, suggesting that these are different behaviors.

Guarding: A series of repeated one-minute time intervals totaling at least five minutes was categorized as guarding behavior, as it was assumed that a bee guarding the entrance would be repeatedly picked up by the reader. If a bee was guarding the entrance, this would cause the reader to be triggered repeatedly and send a new reading every minute The next time interval of at least five minutes indicated the end of a guarding stint, as it was assumed that the bee moved away from the entrance of the colony. NB. It is possible bees recorded in this way were foragers preparing for flight (a decision based on the weather conditions) were exhibiting orientation behavior or were at the nest entrance for an unknown reason other than guarding.

Night 1: When a bee returned to the colony and remained inside until the next morning, or for multiple days, this was categorized as "night 1".

Night 2: When the pattern of "outside" and "inside" indicated that the bee spent the night outside of the colony, this was categorized as "night 2". This was not considered to be foraging as it is impossible to determine how much of this time was actually spent foraging.

When a bee was recorded at a different colony from their own this was categorized as either switching, stealing or visiting, based on the following classifications.

Switched: A bee was considered to have switched colony after it entered a new colony and all subsequent readings were then from that new colony, except in instances of stealing or visiting. When the last two readings from a bee were at a new colony, this was not considered switching, as it cannot be determined if the bee made a permanent change of colony. For the purpose of the statistical analysis, the switching bees' colony was considered to be the colony where it spent the majority of its time.

Stole: When a bee was recorded entering a neighboring colony and re-entering its own colony more than once, and immediately repeating this behavior. To distinguish between deliberately entering a different colony and accidental orientation mistakes, the bee must have entered the new colony at least twice within one day to be considered stealing. Each instance of entering a different colony was classed as one stealing event. On several occasions only one reading from the other colony was picked up, indicating that the bee was missed as it either entered or exited. Therefore, in these cases it was not possible to tell with certainty how long the bee was inside the colony, and so the time intervals of stealing events were not considered when categorizing this behavior.

Visited: Behavior was classed as "visiting" when a bee entered a different colony from its own but did not fulfil the requirements for switching or stealing.
