# Supplementary material for: Investigating the Foraging, Guarding and Drifting Behaviors of Commercial Bombus terrestris
Source: J Insect Behav. 2022 Jan 18;34(5-6):334–45. doi: 10.1007/s10905-021-09790-0 (PMC8813815; doi:10.1007/s10905-021-09790-0)
Supplement: Supplementary file 2 — (PDF 228 kb) [file 10905_2021_9790_MOESM2_ESM.pdf]

Investigating the foraging, guarding and drifting behaviors of commercial *Bombus terrestris*

Journal of Insect Behavior

Ellen L MacKenzie[1], Dave Goulson[1] and Ellen L Rotheray[1]

Affiliations: [1] School of Life Sciences, University of Sussex, Falmer, BN1 9QG, UK

Corresponding Author: Ellen L MacKenzie, ellenmackenzie12@gmail.com

Supplementary Information 2: Final Models and Tables of Coefficients

Final Models

	Model	Model Type	Response Variable	Fixed Effects	Data
a)	1	GLMM	sqrt(longevity)	**mean foraging time** + site + thorax + guard	All
	2		sqrt(longevity)	**forage trips/day** + site + thorax + guard	All
	3		sqrt(mean foraging time)	**thorax***site + forage trips/day	Foragers
	4		sqrt(forage trips/day)	**thorax***site + mean foraging time	Thorax
	5		log(mean foraging time)	**day** + site + colony + thorax	Foragers
	6		forage trips/day	**day***site + thorax + colony + mean foraging time	Foragers
b)	7	GLMM	sqrt(longevity)	**guard** + site + thorax	All
	8		sqrt(mean foraging time)	**guard** + site + forage trips/day	Foragers
	9		sqrt(forage trips/day)	**guard** + site + thorax + mean foraging time	All
	10	GLM	mean guarding time	**thorax** + site + colony	Guards
	11		mean foraging time	**mean guarding time** + site + colony	Guards
	12		sqrt(forage trips/day)	**mean guarding time** + site + colony	Guards
	13	Binomial	guard	**thorax** + colony + longevity + forage trips/day	All
c)	14	GLMM	sqrt(longevity)	**stole** + site + thorax + guard	All
	15		mean foraging time	**stole** + site + thorax + forage trips/day	Foragers
	16		forage trips/day	**stole** + site + thorax + mean foraging time + guard	All
	17	GLM	sqrt(longevity)	**stole** + colony + thorax	Gipps
	18		log(mean foraging time)	**stole** + colony + longevity + thorax + foraging trips/day	Gipps
	19		sqrt(forage trips/day)	**stole** + colony + longevity + thorax + mean foraging time	Gipps
	20	Binomial	stole	**thorax** + colony	All
	21		stole	**thorax** + colony	Gipps
d)	22	GLMM	sqrt(longevity)	**switched***site + thorax + mean foraging time	All
	23		sqrt(mean foraging time)	**switched** + site + forage trips per day	Foragers
	24		sqrt(forage trips/day)	**switched** + site + thorax + mean foraging time	All

Table 2 Models used to analyze a) foraging behavior, b) guarding behavior, c) stealing behavior and d) switching behavior. The models used were general linear models (GLM), general linear mixed models (GLMM), binomial generalized linear models (Binomial) and binomial generalized linear mixed models (Binomial mixed). The square root transformation of the response variable is indicated by *sqrt()* and the log transformation by *log()*. The main effect being investigated in each model is highlighted in bold. The variable *guard* indicates the presence/absence of guarding behavior, *stole* the presence/absence of stealing behavior and *switched* the presence/absence of switching behavior. The data used for each model included either all bees (All), only bees with a known thorax width (Thorax), only bees that exhibited guarding behavior (Guards), only bees that foraged (Foragers) and only bees from colonies placed on Gipps farm (Gipps).

Model Results

Model 1: sqrt(longevity) ~ mean foraging time + site + thorax + guard + (1|colony)

	Estimate	Std. Error	t-value	p-value
Intercept	5.308	2.003	2.649	0.009
Mean foraging time	-0.447	0.249	-1.793	0.076
Site 2	-0.860	0.618	-1.392	0.233
Site 3	-1.144	0.950	-1.204	0.251
Thorax	-0.245	0.355	-0.692	0.491
Guard 1	1.116	0.417	2.676	0.009

Model 2: sqrt(longevity) ~ forage trips/day + site + thorax + guard + (1|colony)

	Estimate	Std. Error	t-value	p-value
Intercept	5.281	2.029	2.603	0.011
Forage trips/day	0.027	0.027	0.996	0.322
Site 2	-0.938	0.553	-1.695	0.162
Site 3	-1.360	0.888	-1.531	0.147
Thorax	-0.344	0.355	-0.967	0.336
Guard 1	1.176	0.424	2.773	0.007

Model 3: sqrt(mean foraging time) ~ thorax*site + forage trips/day + (1|colony)

	Estimate	Std. Error	t-value	p-value
Intercept	1.405	0.477	2.945	0.004
Thorax	-0.050	0.084	-0.597	0.552
Site 2	-1.251	0.601	-2.082	0.040
Site 3	0.615	1.497	0.411	0.683
Forage trips/day	-0.029	0.004	-7.517	<0.001
Site 2:thorax	0.239	0.108	2.208	0.030
Site 3:thorax	-0.067	0.265	-0.252	0.802

Model 4: sqrt(forage trips/day) ~ thorax*site + mean foraging time + (1|colony)

	**Estimate**	**Std. Error**	**t-value**	**p-value**
Intercept	4.704	2.185	2.154	0.034
Thorax	-0.266	0.385	-0.692	0.490
Site 2	-3.482	2.782	-1.252	0.214
Site 3	-0.859	7.042	-0.122	0.903
Mean forage time	-1.285	0.168	-7.650	<0.001
Site 2:thorax	0.716	0.499	1.434	0.155
Site 3:thorax	0.263	1.249	0.210	0.834

Model 5: log(mean foraging time) ~ day + site + colony + thorax + (1|bee)

	**Estimate**	**Std. Error**	**t-value**	**p-value**
Intercept	-1.251	0.66	-1.884	0.063
Day	0.002	0.003	0.620	0.535
Site 2	0.347	0.231	1.504	0.134
Site 3	1.40	0.666	2.111	0.035
Colony 52971	-0.551	0.671	-0.820	0.413
Colony 52972	0.532	0.222	2.392	0.0176
Colony 52973	0.074	0.121	0.610	0.543
Colony 60479	0.062	0.148	0.418	0.677
Colony 60482	0.426	0.230	1.847	0.066
Thorax	0.127	0.108	1.171	0.245

Model 6: forage trips/day ~ day*site + thorax + colony + mean foraging time + (1|bee)

	**Estimate**	**Std. Error**	**t-value**	**p-value**
Intercept	7.999	7.222	1.108	0.271
Day	0.028	0.032	0.878	0.380
Site 2	4.061	2.478	1.638	0.103
Site 3	2.992	6.996	0.428	0.669
Thorax	-0.253	1.181	-0.214	0.831
Colony 52971	5.867	7.268	0.807	0.420
Colony 52972	5.705	2.365	2.413	0.017
Colony 52973	0.115	1.319	0.087	0.931
Colony 60479	0.367	1.603	0.229	0.819
Colony 60482	4.484	2.453	1.828	0.069
Mean forage time	-3.545	0.262	-13.524	<0.001
Site 2:day	0.353	0.070	5.063	<0.001
Site 3:day	-0.811	0.621	-1.3-6	0.192

Model 7: sqrt(longevity) ~ guard + site + thorax + (1|colony)

	**Estimate**	**Std. Error**	**t-value**	**p-value**
Intercept	5.736	2.012	2.851	0.005
Guard 1	1.317	0.429	3.069	0.003
Site 2	-0.911	0.545	-1.671	0.167
Site 3	-1.305	0.899	-1.452	0.166
Thorax	-0.425	0.355	-1.168	0.246

Model 8: sqrt(mean foraging time) ~ guard + site + forage trips/day + (1|colony)

	Estimate	Std. Error	t-value	p-value
Intercept	1.106	0.054	20.452	<0.001
Guard 1	-0.030	0.042	-0.726	0.469
Site 2	0.045	0.072	0.619	0.566
Site 3	0.204	0.089	2.290	0.052
Forage trips/day	-0.026	0.003	-9.209	<0.001

Model 9: sqrt(forage trips/day) ~ guard + site + thorax + mean foraging time + (1|colony)

	Estimate	Std. Error	t-value	p-value
Intercept	2.280	1.364	1.671	0.098
Guard 1	0.347	0.281	0.879	0.382
Site 2	0.416	0.483	0.862	0.435
Site 3	0.575	0.702	0.819	0.431
Thorax	0.153	0.240	0.638	0.525
Mean forage time	-1.216	0.168	-7.226	<0.001

Model 10: mean guarding time ~ thorax + site + colony

	Estimate	Std. Error	t-value	p-value
Intercept	-3.498	2.001	-1.748	0.141
Thorax	0.667	0.367	1.814	0.129
Site 2	0.102	0.235	0.432	0.684
Colony 52973	0.013	0.221	0.060	0.954
Colony 60479	0.273	0.235	1.161	0.298
Colony 60482	-0.028	0.264	-0.107	0.919

Model 11: mean foraging time ~ mean guarding time + site + colony

	Estimate	Std. Error	t-value	p-value
Intercept	0.955	0.114	8.345	<0.001
Mean guard time	-0.059	0.085	-0.696	0.498
Site 2	-0.179	0.189	-0.951	0.358
Colony 52973	0.076	0.178	0.429	0.675
Colony 60479	-0.182	0.186	-0.979	0.344
Colony 60482	0.120	0.167	0.720	0.483

Model 12: sqrt(forage trips/day) ~ mean guarding time + site + colony

	Estimate	Std. Error	t-value	p-value
Intercept	2.761	0.333	8.293	<0.001
Mean guard time	0.111	0.246	0.449	0.660
Site 2	0.388	0.549	0.707	0.491
Colony 52973	-0.332	0.517	-0.642	0.531
Colony 60479	0.600	0.542	1.107	0.287
Colony 60482	-1.307	0.485	-2.693	0.018

Model 13: guard ~ thorax + colony + longevity + forage trips/day

	Estimate	Std. Error	z-value	p-value
Intercept	-24.33	1.028e+04	-0.002	0.998
Thorax	0.895	1.028	0.871	0.384
Colony 52971	-1.789	1.185e+04	0.000	0.100
Colony 52972	12.790	1.028e+04	0.001	0.999
Colony 52973	15.680	1.028e+04	0.002	0.999
Colony 60479	16.260	1.028e+04	0.002	0.999
Colony 60482	13.940	1.028e+04	0.001	0.999
Colony 60583	-1.997	1.052e+04	0.000	0.999
Colony 60595	-2.183	1.075e+04	0.000	0.999
Longevity	0.107	0.042	2.575	0.010
Forage trips/day	0.130	0.065	1.991	0.0464

Model 14: sqrt(longevity) ~ stole + site + thorax + guard + (1|colony)

	**Estimate**	**Std. Error**	**t-value**	**p-value**
Intercept	5.779	2.059	2.806	0.006
Stole 1	0.095	0.840	0.113	0.909
Site 2	-0.908	0.545	-1.668	0.168
Site 3	-1.298	0.901	-1.440	0.169
Thorax	-0.423	0.366	-1.158	0.250
Guard	1.319	0.431	3.057	0.003

Model 15: mean foraging time ~ stole + site + thorax + forage trips/day + (1|colony)

	**Estimate**	**Std. Error**	**t-value**	**p-value**
Intercept	0.121	0.704	0.173	0.863
Stole 1	-0.057	0.281	-0.204	0.839
Site 2	0.128	0.201	0.638	0.556
Site 3	0.476	0.313	1.522	0.152
Thorax	0.217	0.123	1.760	0.082
Forage trips/day	-0.061	0.009	-6.689	<0.001

Model 16: forage trips/day ~ stole + site + thorax + mean foraging time + guard + (1|colony)

	**Estimate**	**Std. Error**	**t-value**	**p-value**
Intercept	7.875	6.546	1.203	0.232
Stole 1	4.331	2.633	1.645	0.103
Site 2	1.784	1.567	1.139	0.316
Site 3	1.989	2.701	0.736	0.471
Thorax	0.369	1.180	0.313	0.755
Mean forage time	-5.087	0.814	-6.250	<0.001
Guard 1	1.510	1.356	1.113	0.268

Model 17: sqrt(longevity) ~ stole + colony + thorax

	Estimate	Std. Error	t-value	p-value
Intercept	11.898	3.745	3.177	0.003
Stole 1	0.360	0.996	0.361	0.720
Colony 60482	-1.349	0.490	-2.755	0.008
Colony 60595	-1.373	0.635	-2.163	0.036
Thorax	-1.361	0.680	-2.001	0.051

Model 18: log(mean foraging time) ~ stole + colony + longevity + thorax + foraging trips/day

	Estimate	Std. Error	t-value	p-value
Intercept	0.138	0.775	0.179	0.859
Stole 1	-0.051	0.170	-0.298	0.768
Colony 60482	-0.246	0.091	-2.714	0.011
Colony 60595	-0.914	0.124	-7.352	<0.001
Longevity	-0.015	0.003	-4.392	<0.001
Thorax	0.107	0.133	0.807	0.426
Forage trips/day	-0.055	0.010	-5.111	<0.001

Model 19: sqrt(forage trips/day) ~ stole + colony + longevity + thorax + mean foraging time

	Estimate	Std. Error	t-value	p-value
Intercept	8.372	2.207	3.794	<0.001
Stole 1	1.046	0.507	2.062	0.047
Colony 60482	-0.523	0.284	-1.842	0.074
Colony 60595	-1.850	0.381	-4.860	<0.001
Longevity	-0.029	0.011	-2,778	0.009
Thorax	-0.763	0.381	-2.002	0.053
Mean forage time	-1.065	0.235	-4.523	<0.001

Model 20: stole ~ thorax + colony

	Estimate	Std. Error	z-value	p-value
Intercept	-62.229	4.608e+04	-0.001	0.999
Thorax	7.779	1.683	1.683	0.092
Colony 52971	-7.431	5.213e+04	0.000	0.100
Colony 52972	15.072	4.608e+04	0.000	0.100
Colony 52973	-4.444	4.671e+04	0.000	0.100
Colony 60479	-4.522	4.733e+04	0.000	0.100
Colony 60482	13.576	4.608+04	0.000	0.100
Colony 60583	-3.779	4.700e+04	0.000	0.100
Colony 60595	-8.233	4.760e+04	0.000	0.100

Model 21: stole ~ thorax + colony

	Estimate	Std. Error	z-value	p-value
Intercept	-47.157	27.236	-1.731	0.083
Thorax	7.779	4.623	1.683	0.092
Colony 60482	-1.496	1.637	-0.914	0.361
Colony 60595	-21.296	4.376e+03	-0.005	0.996

Model 22: sqrt(longevity) ~ switched*site + thorax + mean foraging time + (1|colony)

	Estimate	Std. Error	t-value	p-value
Intercept	6.186	2.130	2.905	0.005
Switched 1	1.014	0.685	1.480	0.142
Site 2	-0.752	0.609	-1.235	0.261
Site 3	-1.143	0.979	-1.168	0.261
Thorax	-0.471	0.382	-1.234	0.220
Mean forage time	-0.192	0.233	-0.822	0.413
Site 2:switched 1	-0.404	0.927	-0.435	0.664

Model 23: sqrt(mean foraging time) ~ switched + site + forage trips per day + (1|colony)

	**Estimate**	**Std. Error**	**t-value**	**p-value**
Intercept	1.106	0.054	20.600	<0.001
Switched 1	-0.014	0.049	-0.289	0.773
Site 2	0.043	0.071	0.609	0.572
Site 3	0.205	0.088	2.315	0.050
Forage trips/day	-0.027	0.003	-9.331	<0.001

Model 24: sqrt(forage trips/day) ~ switched + site + thorax + mean foraging time + (1|colony)

	**Estimate**	**Std. Error**	**t-value**	**p-value**
Intercept	1.590	1.735	0.916	0.362
Switched 1	0.374	0.376	0.995	0.322
Site 2	0.679	0.450	1.509	0.205
Site 3	0.562	0.770	0.729	0.475
Thorax	0.074	0.311	0.239	0.811
Mean forage time	-0.477	0.194	-2.453	0.016

Model 25: switched ~ thorax + longevity + (1|colony)

	**Estimate**	**Std. Error**	**z-value**	**p-value**
Intercept	-5.527	5.063	-1.092	0.275
Thorax	0.498	0.877	0.568	0.570
Longevity	0.033	0.027	1.221	0.222
