# Supplementary material for: Investigating the Foraging, Guarding and Drifting Behaviors of Commercial Bombus terrestris
Source: J Insect Behav. 2022 Jan 18;34(5-6):334–45. doi: 10.1007/s10905-021-09790-0 (PMC8813815; doi:10.1007/s10905-021-09790-0)
Supplement: Supplementary file 3 — (PDF 80 kb) [file 10905_2021_9790_MOESM3_ESM.pdf]

Investigating the foraging, guarding and drifting behaviors of commercial *Bombus terrestris*

Journal of Insect Behavior

Ellen L MacKenzie[1], Dave Goulson[1] and Ellen L Rotheray[1]

Affiliations: [1] School of Life Sciences, University of Sussex, Falmer, BN1 9QG, UK

Corresponding Author: Ellen L MacKenzie, ellenmackenzie12@gmail.com

Supplementary Information 3: Supplementary Figures

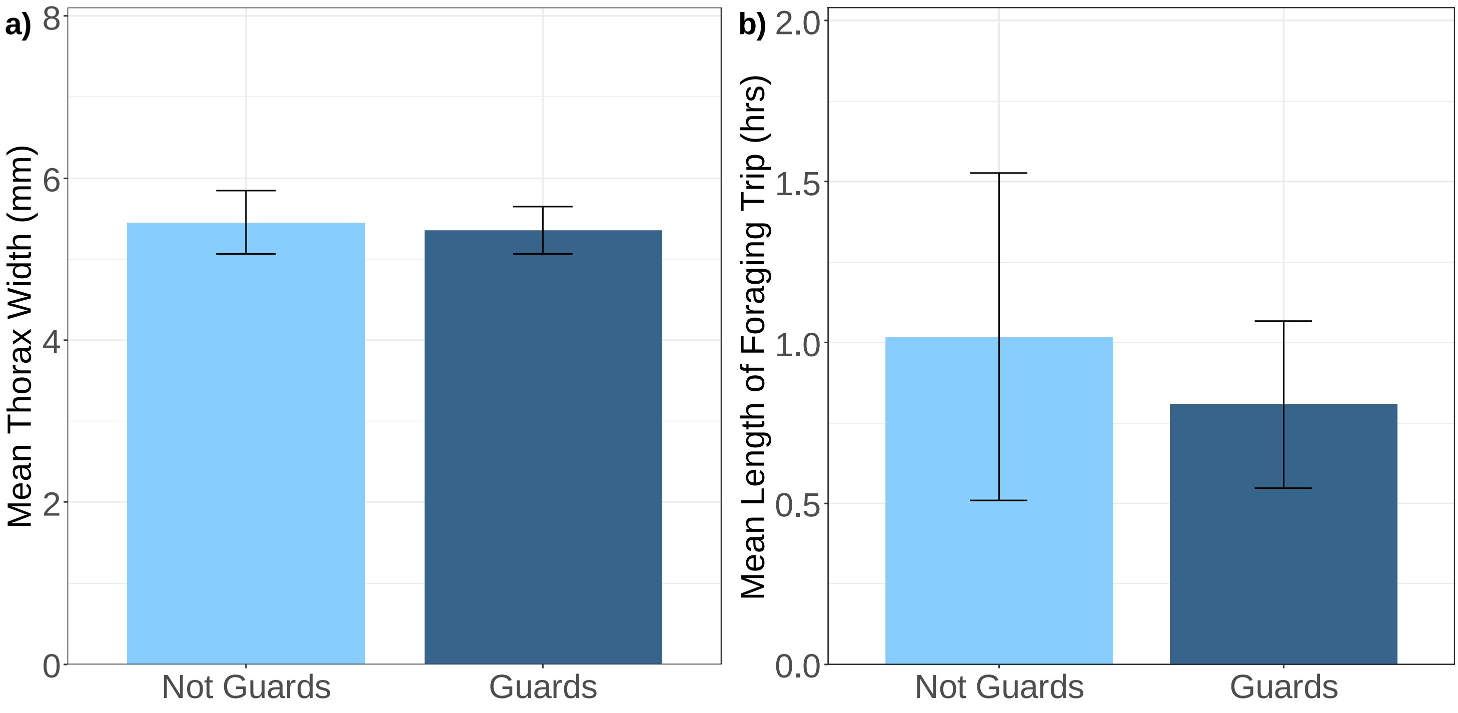

Fig. 1 a) The mean (±SD) thorax width of bees that did not guard and bees that guarded, n = 111. b) The mean (±SD) length of foraging trip for non-guards and guards, n = 159

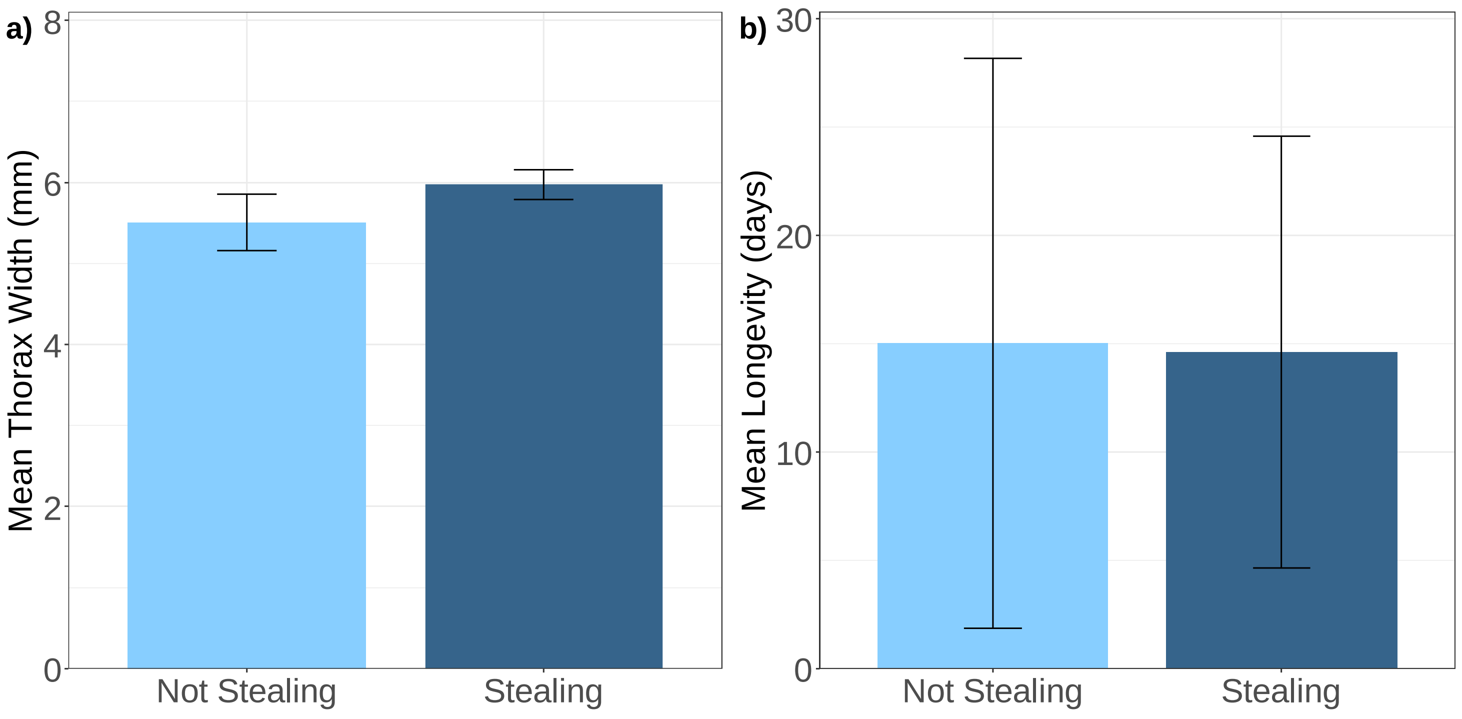

Fig. 2 a) The mean (±SD) thorax width for bees at Gipps farm that were considered to be stealing and bees that did not steal, n = 50. b) The mean (±SD) longevity (days observed within the study) for non-stealing and stealing bees at Gipps, n = 73

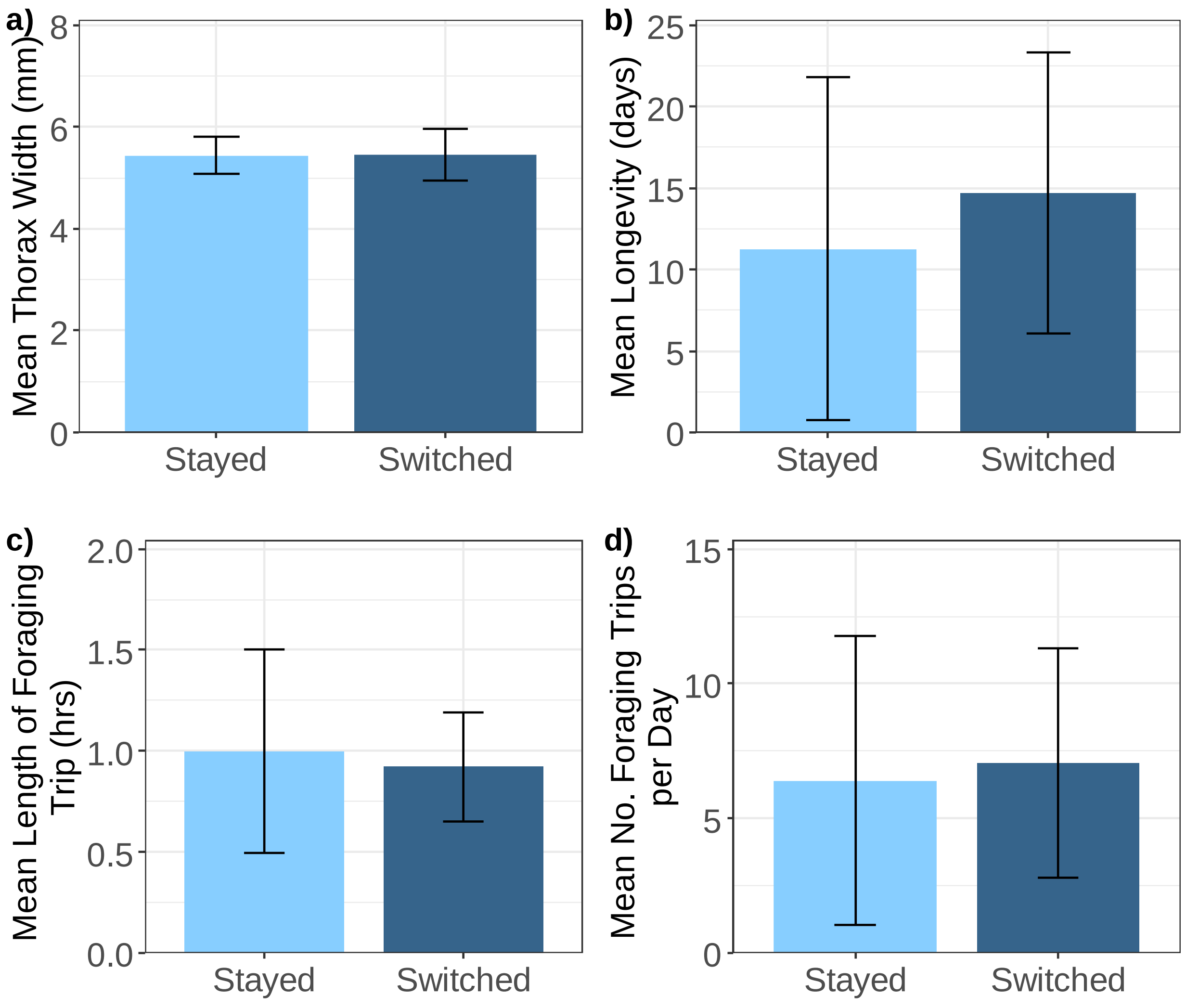

Fig. 3 a) The mean (±SD) thorax width of bees that stayed in their own colony and bees that switched colony, n = 111. b) The mean (±SD) longevity (days observed within the study) for bees that stayed and bees that switched, n = 173. c) The mean (±SD) length of foraging trip for bees that stayed and bees that switched, n = 159. d) The mean (±SD) number of foraging trips per day for bees that stayed and bees that switched, n = 173
