# Supplementary material for: Investigating the Foraging, Guarding and Drifting Behaviors of Commercial Bombus terrestris
Source: J Insect Behav. 2022 Jan 18;34(5-6):334–45. doi: 10.1007/s10905-021-09790-0 (PMC8813815; doi:10.1007/s10905-021-09790-0)
Supplement: Supplementary file 4 — (PDF 213 kb) [file 10905_2021_9790_MOESM4_ESM.pdf]

Investigating the foraging, guarding and drifting behaviors of commercial *Bombus terrestris*

Journal of Insect Behavior

Ellen L MacKenzie[1], Dave Goulson[1] and Ellen L Rotheray[1]

Affiliations: [1] School of Life Sciences, University of Sussex, Falmer, BN1 9QG, UK

Corresponding Author: Ellen L MacKenzie, ellenmackenzie12@gmail.com

Supplementary Information 4: R Code

```
### PACKAGES ###

library(lme4)

library(lmerTest)

library(boot)

library(MuMIn)

library(ggplot2)

library(cowplot)

library(tidyverse)


### DATA ###

## 1.

bees <- read.csv(file.choose())

# file: "bee_behaviour.csv"


bees$bee<-factor(bees$bee)

bees$colony<-factor(bees$colony)

bees$switched<-factor(bees$switched)

bees$stole<-factor(bees$stole)

bees$visit<-factor(bees$visit)

bees$guard<-factor(bees$guard)

bees$site<-factor(bees$site)
```

```
## Subsets
# Foraging bees

foragers <- subset(bees, mean_forage_time > 0)
# Guarding bees
guards <- subset(bees, guard==1)
# Bees with thorax measurement
thorax <- subset(bees, thorax > 0)
# Foragers with thorax measurement
foragersT <- subset(foragers, thorax >0)
# Guards with thorax measurement
guardsT <- subset(guards, thorax >0)
# Bees at Gipps
beesG <- subset(bees, site==1)
# Foragers at Gipps
foragersG <- subset(foragers, site==1)
# Bees with thorax measurement at Gipps
thoraxG <- subset(beesG, thorax>1)

## 2.
days <- read.csv(file.choose())
# File: "foraging_days.csv"

days$site<-factor(days$site)
days$colony<-factor(days$colony)
days$guard<-factor(days$guard)

## Subsets
 foragerdays <- subset(days, mean_forage_time >0)
```

```
### MODELLING ###

## 1. Longevity and Mean foraging time

# Preliminary plots

ggplot(data=foragers,
       aes(x = mean_forage_time, y = longevity,
           colour = site)) +
       geom_point()+
       stat_smooth(method = "lm")


ggplot(data=foragers,
       aes(x = mean_forage_time, y = longevity))+
       geom_point(aes(colour = site))+
       stat_smooth(method = "lm")


# Model selection using AICc

m1 <- lmer(longevity ~ site*mean_forage_time + thorax + guard +(1|colony), data = foragers)

m2 <- lmer(longevity ~ site*mean_forage_time + thorax +(1|colony), data = foragers)

AICc(m1,m2)

# keep m1


m3 <- lmer(longevity ~ site*mean_forage_time + guard +(1|colony), data = foragers)

AICc(m1,m3)

# keep m1


m4 <- lmer(longevity ~ mean_forage_time + site + thorax + guard +(1|colony), data = foragers)

AICc(m1,m4)

# keep m1


m1 <- lmer(longevity ~ site*mean_forage_time + thorax + guard +(1|colony), data = foragers)

# Check assumptions
```

```
par(mfrow=c(2,3))
sresid <- resid(m1, type = "pearson")
hist(sresid)
fits <- fitted(m1)
plot(sresid ~ fits)
qqnorm(resid(m1))
qqline(resid(m1))

## Square root transformation
m1 <- lmer(sqrt(longevity) ~ site*mean_forage_time + thorax + guard +(1|colony), data = foragers)
m2 <- lmer(sqrt(longevity) ~ site*mean_forage_time + thorax +(1|colony), data = foragers)
AICc(m1,m2)
# keep m1

m3 <- lmer(sqrt(longevity) ~ site*mean_forage_time  + guard +(1|colony), data = foragers)
AICc(m1,m3)
# keep m1

m4 <- lmer(sqrt(longevity) ~ mean_forage_time + site + thorax + guard +(1|colony), data = foragers)
AICc(m1,m4)
# keep m4

# Check assumptions
par(mfrow=c(1,3))
sresid <- resid(m4, type = "pearson")
hist(sresid)
 fits <- fitted(m4)
plot(sresid ~ fits)
qqnorm(resid(m4))
qqline(resid(m4))
```

```
# Final Model

model1 <- lmer(sqrt(longevity) ~ mean_forage_time + site + thorax + guard +(1|colony), data = foragers)

summary(model1, ddf="Kenward-Roger")


## 2. Longevity and Foraging Trips/day

# Preliminary plots

ggplot(data=bees,
       aes(x = forage_days, y = longevity,
           colour = site)) +
       geom_point()+
       stat_smooth(method = "lm")


ggplot(data=bees,
       aes(x = forage_days, y = longevity))+
       geom_point(aes(colour = site))+
       stat_smooth(method = "lm")


# Model selection using AICc

m1 <- lmer(longevity ~ site*forage_days + thorax + guard +(1|colony), data = foragers)

m2 <- lmer(longevity ~ site*forage_days + thorax +(1|colony), data = foragers)

AICc(m1,m2)

# keep m1


m3 <- lmer(longevity ~ site*forage_days + guard +(1|colony), data = foragers)

AICc(m1,m3)

# keep m1


m4 <- lmer(longevity ~ forage_days + site + thorax + guard +(1|colony), data = foragers)

AICc(m1,m4)
```

```
# keep m4


# Square root transformation

m5 <- lmer(sqrt(longevity) ~ forage_days + site + thorax + guard +(1|colony), data = foragers)


# Check assumptions

par(mfrow=c(1,3))

sresid <- resid(m5, type = "pearson")

hist(sresid)

fits <- fitted(m5)

plot(sresid ~ fits)

qqnorm(resid(m5))

qqline(resid(m5))


# Final Model

model2 <- lmer(sqrt(longevity) ~ forage_days + site + thorax + guard +(1|colony), data = foragers)

summary(m4, ddf="Kenward-Roger")


## 3. Mean Foraging Time and Thorax

# Preliminary plots

ggplot(data=foragersT,

        aes(x = thorax, y = mean_forage_time,

                colour = site)) +

        geom_point()+

        stat_smooth(method = "lm")

ggplot(data=foragersT,

        aes(x = thorax, y = mean_forage_time))+

        geom_point(aes(colour = site))+

        stat_smooth(method = "lm")
```

```
# Model selection using AICc

m1 <- lmer(mean_forage_time ~ site*thorax + longevity + forage_days + guard + (1|colony), data = foragersT)

m2 <- lmer(mean_forage_time ~ site*thorax + longevity + forage_days + (1|colony), data = foragersT)

AICc(m1,m2)

# keep m2


m3 <- lmer(mean_forage_time ~ site*thorax + longevity + (1|colony), data = foragersT)

 AICc(m2,m3)

# keep m2


m4 <- lmer(mean_forage_time ~ site*thorax + forage_days + (1|colony), data = foragersT)

AICc(m2,m4)

# keep m4


m5 <- lmer(mean_forage_time ~ thorax + site + forage_days + (1|colony), data = foragersT)

AICc(m4,m5)

# keep m4


# Check assumptions

par(mfrow=c(2,3))

sresid <- resid(m4, type = "pearson")

hist(sresid)

fits <- fitted(m4)

plot(sresid ~ fits)

qqnorm(resid(m4))

qqline(resid(m4))


## Square root transformation

m5 <- lmer(sqrt(mean_forage_time) ~ site*thorax + forage_days + (1|colony), data = foragersT)
```

```
# Check assumptions
sresid <- resid(m5, type = "pearson")
hist(sresid)
fits <- fitted(m5)
plot(sresid ~ fits)
qqnorm(resid(m5))
qqline(resid(m5))


# Final Model
model3 <- lmer(sqrt(mean_forage_time) ~ site*thorax + forage_days
+ (1|colony), data = foragersT)
summary(model3, ddf="Kenward-Roger")


## 4. Foraging Trips/day and Thorax
# Preliminary plots
ggplot(data=foragersT,
        aes(x = thorax, y = forage_days,
                colour = site)) +
        geom_point()+
        stat_smooth(method = "lm")


ggplot(data=foragersT,
        aes(x = thorax, y = forage_days))+
        geom_point(aes(colour = site))+
        stat_smooth(method = "lm")


# Model selection using AICc
m1 <- lmer(forage_days ~ site*thorax + longevity + mean_forage_time + guard + (1|colony), data = foragersT)
m2 <- lmer(forage_days ~ site*thorax + longevity + mean_forage_time + (1|colony), data = foragersT)
AICc(m1,m2)
```

```
# keep m1

m3 <- lmer(forage_days ~ site*thorax + longevity + guard + (1|colony), data = foragersT)
AICc(m1,m3)
# keep m1

m4 <- lmer(forage_days ~ site*thorax + mean_forage_time + guard + (1|colony), data = foragersT)
AICc(m1,m4)
# keep m4

m5 <- lmer(forage_days ~ thorax + site + mean_forage_time + guard + (1|colony), data = foragersT)
AICc(m4,m5)
# keep m4

m4 <- lmer(forage_days ~ site*thorax + mean_forage_time + guard + (1|colony), data = foragersT)

# Check assumptions
par(mfrow=c(2,3))
sresid <- resid(m4, type = "pearson")
hist(sresid)
fits <- fitted(m4)
plot(sresid ~ fits)
qqnorm(resid(m4))
qqline(resid(m4))

## Square root transformation
m1 <- lmer(sqrt(forage_days) ~ site*thorax + longevity + mean_forage_time + guard + (1|colony), data =
foragersT)
m2 <- lmer(sqrt(forage_days) ~ site*thorax + longevity + mean_forage_time + (1|colony), data = foragersT)
AICc(m1,m2)
```

```
# keep m2

m3 <- lmer(sqrt(forage_days) ~ site*thorax + longevity + (1|colony), data = foragersT)
AICc(m2,m3)
# keep m2

m4 <- lmer(sqrt(forage_days) ~ site*thorax + mean_forage_time + (1|colony), data = foragersT)
AICc(m3,m4)
# keep m4

m5 <- lmer(sqrt(forage_days) ~ thorax + site + mean_forage_time + (1|colony), data = foragersT)
AICc(m4,m5)
# keep m4

m4 <- lmer(sqrt(forage_days) ~ site*thorax + mean_forage_time + (1|colony), data = foragersT)

# Check assumptions
sresid <- resid(m4, type = "pearson")
hist(sresid)
fits <- fitted(m4)
plot(sresid ~ fits)
qqnorm(resid(m4))
qqline(resid(m4))

# Final Model
model4 <- lmer(sqrt(forage_days) ~ site*thorax + mean_forage_time + (1|colony), data = foragersT)
summary(model4, ddf="Kenward-Roger")

## 5. Mean Foraging Time and Day
# Preliminary plots
```

```
ggplot(data=foragerdays,
	aes(x = day, y = mean_forage_time,
		colour = site)) +
	geom_point()+
	stat_smooth(method = "lm")

ggplot(data=foragerdays,
	aes(x = day, y = mean_forage_time,
	colour = site)) +
	geom_point()+
	stat_smooth(method = "lm")+
	facet_grid(. ~ site)

ggplot(data=foragerdays,
	aes(x = day, y = mean_forage_time))+
	geom_point(aes(colour = site))+
	stat_smooth(method = "lm")

# Model selection using AICc
 m1 <- lmer(mean_forage_time ~ day + site + colony + thorax + longevity + (1|bee), data = foragerdays)
m2 <- lmer(mean_forage_time ~ day + site + colony + thorax + (1|bee), data = foragerdays)
AICc(m1,m2)
# keep m2

m3 <- lmer(mean_forage_time ~ day + site + colony + (1|bee), data = foragerdays)
AICc(m2,m3)
# keep m2

m4 <- lmer(mean_forage_time ~ colony*day + site +  thorax + (1|bee), data = foragerdays)
AICc(m2,m4)
```

```
# keep m2

m5 <- lmer(mean_forage_time ~ site*day + colony + thorax + (1|bee), data = foragerdays)
AICc(m2,m5)
# keep m2

# Check assumptions
par(mfrow=c(2,3))
sresid <- resid(m2, type = "pearson")
hist(sresid)
fits <- fitted(m2)
plot(sresid ~ fits)
qqnorm(resid(m2))
qqline(resid(m2))

# Log transformation
m2a <- lmer(log(mean_forage_time) ~ day + site + colony + thorax + (1|bee), data = foragerdays)

# Check assumptions
sresid <- resid(m2a, type = "pearson")
hist(sresid)
fits <- fitted(m2a)
plot(sresid ~ fits)
qqnorm(resid(m2a))
qqline(resid(m2a))

# Final Model
model5 <- lmer(log(mean_forage_time) ~ day + site + colony + thorax + (1|bee), data = foragerdays)
summary(model5, ddf="Kenward-Roger")
```

```
## 6. Mean Foraging Trips/day and Day

# Preliminary plots

ggplot(data=foragerdays,
        aes(x = day, y = forage_days,
            colour = site)) +
        geom_point()+
        stat_smooth(method = "lm")


ggplot(data=foragerdays,
        aes(x = day, y = forage_days,
        colour = site)) +
        geom_point()+
        stat_smooth(method = "lm")+
        facet_grid(. ~ site)


ggplot(data=foragerdays,
        aes(x = day, y = forage_days))+
        geom_point(aes(colour = site))+
        stat_smooth(method = "lm")


# Model selection using AICc

m1 <- lmer(forage_days ~ site*day + thorax + colony + longevity + mean_forage_time + guard +  (1|bee), data = foragerdays)

m2 <- lmer(forage_days ~ site*day + thorax + colony + longevity + mean_forage_time +  (1|bee), data = foragerdays)

AICc(m1,m2)

# keep m2


m3 <- lmer(forage_days ~ site*day + thorax + colony + longevity + (1|bee), data = foragerdays)

AICc(m2,m3)

# keep m2
```

```r
m4 <- lmer(forage_days ~ site*day + thorax + colony + mean_forage_time +  (1|bee), data = foragerdays)

AICc(m2,m4)

# keep m4


m5 <- lmer(forage_days ~ site*day + thorax + mean_forage_time +  (1|bee), data = foragerdays)

AICc(m4,m5)

# keep m4


m6 <- lmer(forage_days ~ site*day +colony + mean_forage_time +  (1|bee), data = foragerdays)

AICc(m4,m6)

# keep m4


m7 <- lmer(forage_days ~ day + site + thorax + colony + mean_forage_time +  (1|bee), data = foragerdays)

 AICc(m4,m7)

# keep m4


# Check assumptions

sresid <- resid(m4, type = "pearson")

hist(sresid)

fits <- fitted(m4)

plot(sresid ~ fits)

qqnorm(resid(m4))

qqline(resid(m4))


# Final Model

model6 <- lmer(forage_days ~ site*day + thorax + colony + mean_forage_time +  (1|bee), data = foragerdays)

summary(model6, ddf="Kenward-Roger")


## 7. Longevity and Guarding
```

```
# Preliminary plots
gplot(data=bees,
        aes(x = guard, y = longevity)) +
        geom_boxplot()+
stat_summary(fun.y = "mean",
        geom = "point",
        shape = 5, size = 3)

ggplot(data=bees,
        aes(x = guard, y = longevity)) +
        geom_boxplot()+
        facet_grid(. ~ site)+
stat_summary(fun.y = "mean",
        geom = "point",
        shape = 5, size = 3)

# Model selection using AICc
m1 <- lmer(longevity ~ site*guard + thorax + mean_forage_time + forage_days + (1|colony), data = bees)
m2 <- lmer(longevity ~ site*guard + thorax + mean_forage_time + (1|colony), data = bees)
AICc(m1,m2)
## keep m2

m3 <- lmer(longevity ~ site*guard + thorax + (1|colony), data = bees)
AICc(m2,m3)
## keep m2

m4 <- lmer(longevity ~ site*guard  + mean_forage_time + (1|colony), data = bees)
 AICc(m2,m4)
## keep m2
```

```
m5 <- lmer(longevity ~ guard + site + thorax + mean_forage_time + (1|colony), data = bees)

AICc(m2,m5)

## keep m2


m2 <- lmer(longevity ~ site*guard + thorax + mean_forage_time + (1|colony), data = bees)


# Check assumptions

par(mfrow=c(2,3))

sresid <- resid(m2, type = "pearson")

hist(sresid)

fits <- fitted(m2)

plot(sresid ~ fits)

qqnorm(resid(m2))

qqline(resid(m2))


## Square root transformation


m1 <- lmer(sqrt(longevity) ~ site*guard + thorax + mean_forage_time + forage_days + (1|colony), data = bees)

m2 <- lmer(sqrt(longevity) ~ site*guard + thorax + mean_forage_time + (1|colony), data = bees)

AICc(m1,m2)

## keep m2


m3 <- lmer(sqrt(longevity) ~ site*guard + thorax + (1|colony), data = bees)

AICc(m2,m3)

## keep m3


m4 <- lmer(sqrt(longevity) ~ site*guard + (1|colony), data = bees)

AICc(m3,m4)

## keep m3
```

```
m5 <- lmer(sqrt(longevity) ~ guard + site + thorax + (1|colony), data = bees)

AICc(m3,m5)

## keep m5


m5 <- lmer(sqrt(longevity) ~ guard + site + thorax + (1|colony), data = bees)


# Check assumptions

sresid <- resid(m5, type = "pearson")

hist(sresid)

fits <- fitted(m5)

plot(sresid ~ fits)

qqnorm(resid(m5))

qqline(resid(m5))


# Final Model
 model7 <- lmer(sqrt(longevity) ~ guard + site + thorax + (1|colony), data = bees)

summary(model7, ddf="Kenward-Roger")


## 8. Mean foraging time and Guarding

# Preliminary plots

ggplot(data=foragers,

        aes(x = guard, y = mean_forage_time)) +

        geom_boxplot()+

stat_summary(fun.y = "mean",

        geom = "point",

        shape = 5, size = 3)


ggplot(data=foragers,

        aes(x = guard, y = mean_forage_time)) +

        geom_boxplot()+
```

```
    facet_grid(. ~ site)+
stat_summary(fun.y = "mean",
    geom = "point",
    shape = 5, size = 3)

# Model selection using AICc
m1 <- lmer(mean_forage_time ~ site*guard + longevity + thorax + forage_days + (1|colony), data = foragers)
m2 <- lmer(mean_forage_time ~ site*guard + longevity + thorax + (1|colony), data = foragers)
AICc(m1,m2)
# keep m1

m3 <- lmer(mean_forage_time ~ site*guard + longevity + forage_days + (1|colony), data = foragers)
AICc(m1,m3)
# keep m1

m4 <- lmer(mean_forage_time ~ site*guard + thorax + forage_days + (1|colony), data = foragers)
AICc(m1,m4)
# keep m4

m5 <- lmer(mean_forage_time ~ guard + site + thorax + forage_days + (1|colony), data = foragers)
AICc(m4,m5)
# keep m5

m5 <- lmer(mean_forage_time ~ guard + site + thorax + forage_days + (1|colony), data = foragers)

# Check assumptions
par(mfrow=c(2,3))
sresid <- resid(m5, type = "pearson")
hist(sresid)
```

```
fits <- fitted(m5)

plot(sresid ~ fits)

qqnorm(resid(m5))

qqline(resid(m5))


# Square root transformation

m1 <- lmer(sqrt(mean_forage_time) ~ site*guard + longevity + thorax + forage_days + (1|colony), data =
foragers)

m2 <- lmer(sqrt(mean_forage_time) ~ site*guard + longevity + thorax + (1|colony), data = foragers)

AICc(m1,m2)

# keep m1


m3 <- lmer(sqrt(mean_forage_time) ~ site*guard + longevity  + forage_days + (1|colony), data = foragers)

AICc(m1,m3)

# keep m3


m4 <- lmer(sqrt(mean_forage_time) ~ site*guard + forage_days + (1|colony), data = foragers)

 AICc(m3,m4)

# keep m4


m5 <- lmer(sqrt(mean_forage_time) ~ guard + site + forage_days + (1|colony), data = foragers)

AICc(m4,m5)

# keep m5


m5 <- lmer(sqrt(mean_forage_time) ~ guard + site + forage_days + (1|colony), data = foragers)


# Check assumptions

sresid <- resid(m5, type = "pearson")

hist(sresid)
```

```
fits <- fitted(m5)

plot(sresid ~ fits)

qqnorm(resid(m5))

qqline(resid(m5))


# Final Model

model8 <- lmer(sqrt(mean_forage_time) ~ guard + site + forage_days + (1|colony), data = foragers)

summary(model8,ddf="Kenward-Roger")


## 9. Foraging trips/day and Guarding

# Preliminary plots

ggplot(data=foragers,

        aes(x = guard, y = forage_days)) +

        geom_boxplot()+

stat_summary(fun.y = "mean",

        geom = "point",

        shape = 5, size = 3)


ggplot(data=foragers,

        aes(x = guard, y = forage_days)) +

        geom_boxplot()+

        facet_grid(. ~ site)+

stat_summary(fun = "mean",

        geom = "point",

        shape = 5, size = 3)


# Model selection using AICc

m1 <- lmer(forage_days ~ site*guard + longevity + thorax + mean_forage_time + (1|colony), data = foragers)

m2 <- lmer(forage_days ~ site*guard + longevity + thorax + (1|colony), data = foragers)

AICc(m1,m2)
```

```
# keep m1

m3 <- lmer(forage_days ~ site*guard + longevity + mean_forage_time + (1|colony), data = foragers)
AICc(m1,m3)
# keep m1

m4 <- lmer(forage_days ~ site*guard + thorax + mean_forage_time + (1|colony), data = foragers)
AICc(m1,m4)
# keep m4

m5 <- lmer(forage_days ~ site*guard + mean_forage_time + (1|colony), data = foragers)
AICc(m4,m5)
# keep m4

m6 <- lmer(forage_days ~ guard + site + thorax + mean_forage_time + (1|colony), data = foragers)
AICc(m4,m6)
# keep m4

m4 <- lmer(forage_days ~ site*guard + thorax + mean_forage_time + (1|colony), data = foragers)

# Check assumptions
par(mfrow=c(2,3))
sresid <- resid(m4, type = "pearson")
hist(sresid)

fits <- fitted(m4)
plot(sresid ~ fits)
qqnorm(resid(m4))
qqline(resid(m4))
```

```
# Square root transformation

m4a <- lmer(sqrt(forage_days) ~ site*guard + thorax + mean_forage_time + (1|colony), data = foragers)


# Check assumptions

sresid <- resid(m4a, type = "pearson")

hist(sresid)

fits <- fitted(m4a)

plot(sresid ~ fits)

qqnorm(resid(m4a))

qqline(resid(m4a))


# Final Model

model9 <- lmer(sqrt(forage_days) ~ site*guard + thorax + mean_forage_time + (1|colony), data = foragers)

summary(model9, ddf="Kenward-Roger")


## 10. Mean guarding time and Thorax

# Preliminary plots

ggplot(data=guards,

        aes(x = thorax, y = mean_guard_time,

                colour = site)) +

        geom_point()+

        stat_smooth(method = "lm")


ggplot(data=guards,

        aes(x = thorax, y = mean_guard_time))+

        geom_point(aes(colour = site))+

        stat_smooth(method = "lm")


# Preliminary plots with outlier removed

ggplot(data=guards2,
```

```
    aes(x = thorax, y = mean_guard_time,
        colour = site)) +
    geom_point()+
    stat_smooth(method = "lm")


ggplot(data=guards2,
    aes(x = thorax, y = mean_guard_time))+
    geom_point(aes(colour = site))+
    stat_smooth(method = "lm")


# Model selection using AICc
m1 <- lm(mean_guard_time ~ site*thorax + colony + longevity + forage_days+ mean_forage_time, data = guardsT)
m2 <- lm(mean_guard_time ~ site*thorax + colony + longevity + forage_days, data = guardsT)
AICc(m1,m2)
# keep m2


m3 <- lm(mean_guard_time ~ site*thorax + colony + longevity, data = guardsT)
AICc(m2,m3)
# keep m3


m4 <- lm(mean_guard_time ~ site*thorax + colony , data = guardsT)
AICc(m3,m4)
# keep m4


m5 <- lm(mean_guard_time ~ thorax + site + colony , data = guardsT)
AICc(m4,m5)
# keep m5


# Check assumptions
```

```
par(mfrow=c(2,2))

plot(m5)

# Remove outlier

guards2 <- subset(guardsT, mean_guard_time < 0.9)

# Check assumptions

m5 <- lm(mean_guard_time ~ thorax + site + colony , data = guards2)

plot(m5)


# Final Model

model10 <- lm(mean_guard_time ~ thorax + site + colony, data = guards2)

summary(model10)


## 11. Mean foraging time and Mean guarding time

# Preliminary plots

ggplot(data=guards,

        aes(x = mean_guard_time, y = mean_forage_time,

                colour = site)) +

        geom_point()+

        stat_smooth(method = "lm")


ggplot(data=guards,

        aes(x = mean_guard_time, y = mean_forage_time))+

        geom_point(aes(colour = site))+

        stat_smooth(method = "lm")


# Model selection using AICc

m1 <- lm(mean_forage_time ~ site*mean_guard_time + colony + longevity + thorax + forage_days, data =
guards)

m2 <- lm(mean_forage_time ~ site*mean_guard_time + colony + longevity + thorax , data = guards)

AICc(m1,m2)
```

```
# keep m2

m3 <- lm(mean_forage_time ~ site*mean_guard_time + colony + longevity , data = guards)
AICc(m2,m3)
# keep m3

m4 <- lm(mean_forage_time ~ site*mean_guard_time + colony , data = guards)
AICc(m3,m4)
# keep m4

m5 <- lm(mean_forage_time ~ mean_guard_time + site + colony, data = guards)
AICc(m4,m5)
# keep m5

# Check assumptions
par(mfrow=c(2,2))
plot(m5)

# Final Model
model11 <- lm(mean_forage_time ~ mean_guard_time + site + colony, data = guards)
summary(model11)

## 12. Foraging trips/day and Mean guarding time
# Preliminary plots
ggplot(data=guards,
        aes(x = mean_guard_time, y = forage_days,
                colour = site)) +
        geom_point()+
        stat_smooth(method = "lm")
```

```
ggplot(data=guards,
	aes(x = mean_guard_time, y = forage_days))+
	geom_point(aes(colour = site))+
	stat_smooth(method = "lm")

# Model selection using AICc
m1 <- lm(forage_days ~ site*mean_guard_time + colony + longevity + thorax + mean_forage_time, data = guards)
m2 <- lm(forage_days ~ site*mean_guard_time + colony + longevity + thorax , data = guards)
AICc(m1,m2)
# keep m2

m3 <- lm(forage_days ~ site*mean_guard_time + colony + longevity  , data = guards)
AICc(m2,m3)
# keep m3

m4 <- lm(forage_days ~ site*mean_guard_time + colony  , data = guards)
 AICc(m3,m4)
# keep m4

m5 <- lm(forage_days ~ mean_guard_time + site + colony  , data = guards)
AICc(m4,m5)
# keep m5

# Check assumptions
par(mfrow=c(2,2))
plot(m5)

# Square root transformation
m5a <- lm(sqrt(forage_days) ~ mean_guard_time + site + colony , data = guards)
```

```
plot(m5a)


# Final Model

model12 <- lm(sqrt(forage_days) ~ mean_guard_time + site + colony  , data = guards)

summary(model12)


## 13. Guarding and Thorax

# Preliminary plots

ggplot(data=thorax,

       aes(x = guard, y = thorax)) +

       geom_boxplot()+

       stat_summary(fun.y = "mean",

       geom = "point",

       shape = 5, size = 3)


ggplot(data=thorax,

       aes(x = guard, y = thorax)) +

       geom_boxplot()+

       facet_grid(. ~ site)+

stat_summary(fun.y = "mean",

       geom = "point",

       shape = 5, size = 3)


# Model selection using AICc

m1 <- glm(guard ~ thorax*colony + longevity + forage_days + mean_forage_time, family = binomial
(link="cloglog"), data=thorax)

m2 <- glm(guard ~ thorax*colony + longevity + forage_days, family = binomial (link="cloglog"), data=thorax)

AICc(m1,m2)

# keep m2
```

```
m3 <- glm(guard ~ thorax*colony + longevity, family = binomial (link="cloglog"), data=thorax)

AICc(m2,m3)

# keep m2


m4 <- glm(guard ~ thorax*colony + forage_days, family = binomial (link="cloglog"), data=thorax)

AICc(m2,m4)

# keep m2


m5 <- glm(guard ~ thorax + colony + longevity + forage_days, family = binomial (link="cloglog"),
data=thorax)

AICc(m2,m5)

# keep m5


# Check assumptions

 glm.diag.plots(m5)


# Remove outliers

row.names(thorax) <- 1:nrow(thorax)

thorax2 <- thorax[-c(68,70,80),]


m5a <- glm(guard ~ thorax + colony + longevity + forage_days, family = binomial (link="cloglog"),
data=thorax2)


# Check assumptions

glm.diag.plots(m5a)

devresid <- resid(m5a, type="deviance")

hist(devresid)


# Final Model

model13 <- glm(guard ~ thorax + colony + longevity + forage_days, family = binomial (link="cloglog"),
data=thorax2)
```

```
summary(model13)


## 14. Longevity and Stealing (all bees)

# Preliminary plots

ggplot(data=bees,

        aes(x = stole, y = longevity)) +

        geom_boxplot()+

stat_summary(fun.y = "mean",

        geom = "point",

        shape = 5, size = 3)


# Model selection using AICc

 m1 <- lmer(longevity ~ stole + site + thorax + mean_forage_time + forage_days + guard + (1|colony), data =
bees)

m2 <- lmer(longevity ~ stole + site + thorax + mean_forage_time + forage_days +(1|colony), data = bees)

AICc(m1,m2)

# keep m1


m3 <- lmer(longevity ~ stole + site + thorax + mean_forage_time + guard + (1|colony), data = bees)


AICc(m1,m3)

# keep m3


m4 <- lmer(longevity ~ stole + site + thorax + guard + (1|colony), data = bees)

AICc(m3,m4)

# keep m4


m5 <- lmer(longevity ~ stole + site + guard + (1|colony), data = bees)

AICc(m4,m5)

# keep m4
```

```
m6 <- lmer(longevity ~ site*stole  + thorax + guard + (1|colony), data = bees)

AICc(m4,m6)

# keep m4


# Check assumptions

par(mfrow=c(2,3))

sresid <- resid(m4, type = "pearson")

hist(sresid)

fits <- fitted(m4)

plot(sresid ~ fits)

 qqnorm(resid(m4))

qqline(resid(m4))


# Square root transformation

m4a <- lmer(sqrt(longevity) ~ stole + site + thorax + guard + (1|colony), data = bees)


# Check assumptions

sresid <- resid(m4a, type = "pearson")

hist(sresid)

fits <- fitted(m4a)

plot(sresid ~ fits)

qqnorm(resid(m4a))

qqline(resid(m4a))


# Final Model

model14 <- lmer(sqrt(longevity) ~ stole + site + thorax + guard + (1|colony), data = bees)

summary(model14, ddf="Kenward-Roger")


## 15. Mean foraging time and Stealing (all bees)
```

```
# Preliminary plots

plot(data=foragers,

        aes(x = stole, y = mean_forage_time)) +

        geom_boxplot()+

        stat_summary(fun.y = "mean",

        geom = "point",

        shape = 5, size = 3)


# Model selection using AICc

m1 <- lmer(mean_forage_time ~ stole + site + longevity + thorax + forage_days + guard + (1|colony), data =
foragers)

m2 <- lmer(mean_forage_time ~ stole + site + longevity + thorax + forage_days + (1|colony), data = foragers)

AICc(m1,m2)

# keep m2


m3 <- lmer(mean_forage_time ~ stole + site + longevity + thorax + (1|colony), data = foragers)

AICc(m2,m3)

# keep m2


m4 <- lmer(mean_forage_time ~ stole + site + longevity  +forage_days + (1|colony), data = foragers)

AICc(m2,m4)

# keep m2


m5 <- lmer(mean_forage_time ~ stole + site + thorax +forage_days + (1|colony), data = foragers)

AICc(m2,m5)

# keep m5


# Check assumptions

par(mfrow=c(2,3))

sresid <- resid(m5, type = "pearson")
```

```
hist(sresid)

fits <- fitted(m5)

plot(sresid ~ fits)

qqnorm(resid(m5))

qqline(resid(m5))


# Final Model

model15 <- lmer(mean_forage_time ~ stole + site + thorax + forage_days + (1|colony), data = foragers)

summary(model15, ddf="Kenward-Roger")


## 16. Foraging trips/day and Stealing (all bees)

# Preliminary plots

ggplot(data=foragers,

        aes(x = stole, y = forage_days)) +

        geom_boxplot()+

        stat_summary(fun.y = "mean",

        geom = "point",

        shape = 5, size = 3)


# Model selection using AICc

m1 <- lmer(forage_days ~ stole + site + longevity + thorax + mean_forage_time + guard + (1|colony), data =
foragers)

m2 <- lmer(forage_days ~ stole + site + longevity + thorax + mean_forage_time + (1|colony), data = foragers)

AICc(m1,m2)

# keep m1


m3 <- lmer(forage_days ~ stole + site + longevity + thorax + guard + (1|colony), data = foragers)

AICc(m1,m3)

# keep m1
```

```
m4 <- lmer(forage_days ~ stole + site + longevity  + mean_forage_time + guard + (1|colony), data = foragers)

AICc(m1,m4)

# keep m1


m5 <- lmer(forage_days ~ stole + site + thorax + mean_forage_time + guard + (1|colony), data = foragers)

AICc(m1,m5)

# keep m5


# Model assumptions

par(mfrow=c(2,3))

sresid <- resid(m5, type = "pearson")

hist(sresid)

fits <- fitted(m5)

plot(sresid ~ fits)

qqnorm(resid(m5))

qqline(resid(m5))


# Final Model

model16 <- lmer(forage_days ~ stole + site + thorax + mean_forage_time + guard + (1|colony), data = foragers)

summary(model16, ddf="Kenward-Roger")


## 17. Longevity and Stealing (Gipps)

# Preliminary plots

ggplot(data=beesG,

        aes(x = stole, y = longevity)) +

        geom_boxplot()+

        stat_summary(fun.y = "mean",

        geom = "point",

        shape = 5, size = 3)
```

```
# Model selection using AICc

m1 <- lm(longevity ~ stole + colony + thorax + mean_forage_time + forage_days + guard, data = beesG)

 m2 <- lm(longevity ~ stole + colony + thorax + mean_forage_time + forage_days, data = beesG)

AICc(m1,m2)

# keep m2


m3 <- lm(longevity ~ stole + colony + thorax + mean_forage_time, data = beesG)

AICc(m2,m3)

# keep m3


m4 <- lm(longevity ~ stole + colony + thorax , data = beesG)

AICc(m3,m4)

# keep m4


m5 <- lm(longevity ~ stole + colony  , data = beesG)

AICc(m4,m5)

# keep m4


m6 <- lm(longevity ~ stole + thorax , data = beesG)

AICc(m4,m6)

# keep m4


m7 <- lm(longevity ~ colony*stole +thorax , data = beesG)

AICc(m4,m7)

# keep m4


# Check assumptions

plot(m4)


# Square root transformation
```

```
 m4a <- lm(sqrt(longevity) ~ stole + colony + thorax , data = beesG)
# Check assumptions
plot(m4a)

# Final Model
model17 <- lm(sqrt(longevity) ~ stole + colony + thorax , data = beesG)
summary(model17)

## 18. Mean foraging time and Stealing (Gipps)
# Preliminary plots
ggplot(data=foragersG,
        aes(x = stole, y = mean_forage_time)) +
        geom_boxplot()+
        stat_summary(fun.y = "mean",
        geom = "point",
        shape = 5, size = 3)

# Model selection using AICc
m1 <- lm(mean_forage_time ~ stole + colony + longevity + thorax + forage_days + guard , data = foragersG)
m2 <- lm(mean_forage_time ~ stole + colony + longevity + thorax + forage_days, data = foragersG)
AICc(m1,m2)
# keep m2

m3 <- lm(mean_forage_time ~ stole + colony + longevity + thorax , data = foragersG)
AICc(m2,m3)
# keep m2

m4 <- lm(mean_forage_time ~ stole + colony + longevity+ forage_days, data = foragersG)
AICc(m2,m4)
# keep m2
```

```
m5 <- lm(mean_forage_time ~ stole + colony  + thorax + forage_days, data = foragersG)

AICc(m2,m5)

# keep m2


m6 <- lm(mean_forage_time ~ colony*stole + longevity + thorax + forage_days, data = foragersG)

AICc(m2,m6)

# keep m2


# Check assumptions

par(mfrow=c(2,2))

plot(m2)


# Log transformation

m2a <- lm(log(mean_forage_time) ~ stole + colony + longevity + thorax + forage_days, data = foragersG)


# Check assumptions

plot(m2a)


# Remove outliers

foragersG2 <- subset(foragersG, bee != "7B 17 66 01 0B 00 12 E0" & bee != "7A 17 66 01 0B 00 12 E0" & bee
!= "8D 17 66 01 0B 00 12 E0")


model2b <- lm(log(mean_forage_time) ~ stole + colony + longevity + thorax + forage_days, data = foragersG2)


# Check assumptions

 plot(model2b)

# Final Model

model18 <- lm(log(mean_forage_time) ~ stole + colony + longevity + thorax + forage_days, data = foragersG2)

summary(model18)
```

```
## 19. Foraging trips/day and Stealing (Gipps)
# Preliminary plots
ggplot(data=foragersG,
        aes(x = stole, y = forage_days)) +
        geom_boxplot()+
        stat_summary(fun.y = "mean",
        geom = "point",
        shape = 5, size = 3)

ggplot(data=foragersG,
        aes(x = stole, y = forage_days)) +
        geom_boxplot()+
        facet_grid(. ~ colony)+
stat_summary(fun.y = "mean",
        geom = "point",
        shape = 5, size = 3)

# Model selection using AICc
m1 <- lm(forage_days ~ stole + colony + longevity + thorax + mean_forage_time + guard, data = foragersG)
m2 <- lm(forage_days ~ stole + colony + longevity + thorax + mean_forage_time , data = foragersG)
AICc(m1,m2)
# keep m2

m3 <- lm(forage_days ~ stole + colony + longevity + thorax , data = foragersG)
AICc(m2,m3)
# keep m2

m4 <- lm(forage_days ~ stole + colony + longevity + mean_forage_time , data = foragersG)
AICc(m2,m4)
```

```
# keep m2

m5 <- lm(forage_days ~ stole + colony + thorax + mean_forage_time , data = foragersG)
AICc(m2,m5)
# keep m2

m6 <- lm(forage_days ~ stole + longevity + thorax + mean_forage_time , data = foragersG)
AICc(m2,m6)
# keep m2

m7 <- lm(forage_days ~ colony*stole + longevity + thorax + mean_forage_time , data = foragersG)
AICc(m2,m7)
# keep m2

# Check assumptions
plot(m2)

# Square root transformation
m2a <- lm(sqrt(forage_days) ~ stole + colony + longevity + thorax + mean_forage_time , data = foragersG)

# Check assumptions
par(mfrow=c(2,2))
plot(m2a)

# Final Model
model19 <- lm(sqrt(forage_days) ~ stole + colony + longevity + thorax + mean_forage_time , data = foragersG)
summary(model19)

## 20. Stealing and Thorax (all bees)
# Preliminary plots
```

```
ggplot(data=thorax,
        aes(x = stole, y = thorax)) +
        geom_boxplot()+
        stat_summary(fun = "mean",
        geom = "point",
        shape = 5, size = 3)


# Model selection using AICc

m1 <- glm(stole ~ thorax + colony + forage_days + longevity + mean_forage_time ,family = binomial
(link="cloglog"), data=thorax)

m2 <- glm(stole ~ thorax + colony + forage_days + longevity ,family = binomial (link="cloglog"), data=thorax)

AICc(m1,m2)

# keep m2


m3 <- glm(stole ~ thorax + colony + forage_days,family = binomial (link="cloglog"), data=thorax)

AICc(m2,m3)

# keep m3


m4 <- glm(stole ~ thorax + colony,family = binomial (link="cloglog"), data=thorax)

AICc(m3,m4)

# keep m4 (m3 errors)


# Check assumptions

glm.diag.plots(m4)

devresid <- resid(m4, type="deviance")

hist(devresid)


# Final Model

model20 <- glm(stole ~ thorax + colony,family = binomial (link="cloglog"), data=thorax)

summary(model20)
```

21. Stealing and Thorax (Gipps)

```
# Preliminary plots

ggplot(data=thoraxG,
        aes(x = stole, y = thorax)) +
        geom_boxplot()+
        stat_summary(fun = "mean",
        geom = "point",
        shape = 5, size = 3)


# Model selection using AICc

m1 <- glm(stole ~ thorax + colony + mean_forage_time + forage_days + longevity,family = binomial (link="cloglog"), data=thoraxG)

m2 <- glm(stole ~ thorax + colony + mean_forage_time + forage_days,family = binomial (link="cloglog"), data=thoraxG)

AICc(m1,m2)

# keep m2


m3 <- glm(stole ~ thorax + colony + mean_forage_time ,family = binomial (link="cloglog"), data=thoraxG)

 AICc(m2,m3)

# keep m3 (m2 error)


m4 <- glm(stole ~ thorax + colony ,family = binomial (link="cloglog"), data=thoraxG)

AICc(m3,m4)

# keep m4


# Check assumptions

glm.diag.plots(m4)

devresid <- resid(m4, type="deviance")

hist(devresid)
```

```
# Final Model

model21 <- glm(stole ~ thorax + colony,family = binomial (link="cloglog"), data=thoraxG)

summary(model21)


## 22. Longevity and Switched colony

# Preliminary plots

ggplot(data=bees,

        aes(x = switched, y = longevity)) +

        geom_boxplot()+

        stat_summary(fun.y = "mean",

        geom = "point",

        shape = 5, size = 3)


ggplot(data=bees,

        aes(x = switched, y = longevity)) +

        geom_boxplot()+

        facet_grid(. ~ site)+

stat_summary(fun.y = "mean",

        geom = "point",

        shape = 5, size = 3)


# Model selection using AICc

m1 <- lmer(longevity ~ site*switched + thorax + mean_forage_time + forage_days+ (1|colony), data = bees)

m2 <- lmer(longevity ~ site*switched + thorax + mean_forage_time + (1|colony), data = bees)

AICc(m1,m2)

# keep m2


m3 <- lmer(longevity ~ site*switched + thorax  + (1|colony), data = bees)

AICc(m2,m3)

# keep m2
```

```
m4 <- lmer(longevity ~ site*switched + mean_forage_time + (1|colony), data = bees)

AICc(m2,m4)

# keep m2


m5 <- lmer(longevity ~ switched + site + thorax + mean_forage_time + (1|colony), data = bees)

AICc(m2,m5)

# keep m2


# Check assumptions

par(mfrow=c(2,3))

sresid <- resid(m2, type = "pearson")

hist(sresid)

fits <- fitted(m2)

plot(sresid ~ fits)

qqnorm(resid(m2))

qqline(resid(m2))


# Square root transformation

m2a <- lmer(sqrt(longevity) ~ site*switched + thorax + mean_forage_time + (1|colony), data = bees)


# Check assumptions

sresid <- resid(m2a, type = "pearson")

hist(sresid)

fits <- fitted(m2a)

plot(sresid ~ fits)

qqnorm(resid(m2a))

qqline(resid(m2a))


# Final Model
```

```
model22 <- lmer(sqrt(longevity) ~ site*switched + thorax + mean_forage_time + (1|colony), data = bees)


## 23. Mean foraging time and Switched colony

# Preliminary plots

ggplot(data=foragers,
        aes(x = switched, y = mean_forage_time)) +
        geom_boxplot()+
        stat_summary(fun.y = "mean",
        geom = "point",
        shape = 5, size = 3)


ggplot(data=foragers,
        aes(x = switched, y = mean_forage_time)) +
        geom_boxplot()+
        facet_grid(. ~ site)+
stat_summary(fun.y = "mean",
        geom = "point",
        shape = 5, size = 3)


# Model selection using AICc

m1 <- lmer(mean_forage_time ~ site*switched + thorax + longevity + forage_days + guard + (1|colony), data=foragers)

m2 <- lmer(mean_forage_time ~ site*switched + thorax + longevity + forage_days + (1|colony), data=foragers)

AICc(m1,m2)

# keep m2


m3 <- lmer(mean_forage_time ~ site*switched + thorax + longevity  + (1|colony), data=foragers)

AICc(m2,m3)

# keep m2
```

```
m4 <- lmer(mean_forage_time ~ site*switched + thorax + forage_days + (1|colony), data=foragers)

AICc(m2,m4)

# keep m4


m5 <- lmer(mean_forage_time ~ site*switched + forage_days + (1|colony), data=foragers)

AICc(m4,m5)

# keep m4


m6 <- lmer(mean_forage_time ~ switched + site + thorax + forage_days + (1|colony), data=foragers)

AICc(m4,m6)

# keep m6


# Check assumptions

par(mfrow=c(3,3))

 sresid <- resid(m6, type = "pearson")

hist(sresid)

fits <- fitted(m6)

plot(sresid ~ fits)

qqnorm(resid(m6))

qqline(resid(m6))


# Square root transformation

m1 <- lmer(sqrt(mean_forage_time) ~ site*switched + thorax + longevity + forage_days + guard + (1|colony),
data=foragers)

m2 <- lmer(sqrt(mean_forage_time) ~ site*switched + thorax + longevity + forage_days + (1|colony),
data=foragers)

AICc(m1,m2)

# keep m2


m3 <- lmer(sqrt(mean_forage_time) ~ site*switched + thorax + forage_days + (1|colony), data=foragers)

AICc(m2,m3)
```

```
# keep m3


m4 <- lmer(sqrt(mean_forage_time) ~ site*switched + forage_days + (1|colony), data=foragers)

AICc(m3,m4)

# keep m4


m5 <- lmer(sqrt(mean_forage_time) ~ switched + site + forage_days + (1|colony), data=foragers)

AICc(m4,m5)

# keep m5


m5 <- lmer(sqrt(mean_forage_time) ~ switched + site + forage_days + (1|colony), data=foragers)


# Check assumptions

sresid <- resid(m5, type = "pearson")

hist(sresid)

fits <- fitted(m5)

plot(sresid ~ fits)

qqnorm(resid(m5))

qqline(resid(m5))


# Final Model

 model23 <- lmer(sqrt(mean_forage_time) ~ switched + site + forage_days + (1|colony), data=foragers)

 summary(model23, ddf="Kenward-Roger")


## 24. Foraging trips/day and Switched colony

# Preliminary plots

 ggplot(data=bees,

		aes(x = switched, y = forage_days)) +

		geom_boxplot()+

		stat_summary(fun.y = "mean",
```

```
        geom = "point",
        shape = 5, size = 3)

ggplot(data=bees,
        aes(x = switched, y = forage_days)) +
        geom_boxplot()+
        facet_grid(. ~ site)+
stat_summary(fun.y = "mean",
        geom = "point",
        shape = 5, size = 3)

# Model selection using AICc
m1 <- lmer(forage_days ~ site*switched + thorax + longevity + mean_forage_time + guard + (1|colony),
data=foragers)
m2 <- lmer(forage_days ~ site*switched + thorax + longevity + mean_forage_time + (1|colony), data=foragers)
AICc(m1,m2)
# keep m1

m3 <- lmer(forage_days ~ site*switched + thorax + longevity + guard + (1|colony), data=foragers)
AICc(m1,m3)
# keep m1

m4 <- lmer(forage_days ~ site*switched + thorax + mean_forage_time + guard + (1|colony), data=foragers)
AICc(m1,m4)
# keep m4

m5 <- lmer(forage_days ~ site*switched + mean_forage_time + guard + (1|colony), data=foragers)
AICc(m4,m5)
# keep m4
```

```
m6 <- lmer(forage_days ~ switched + site + thorax + mean_forage_time + guard + (1|colony), data=foragers)

AICc(m4,m6)

# keep m4


m4 <- lmer(forage_days ~ site*switched + thorax + mean_forage_time + guard + (1|colony), data=foragers)


# Check assumptions

par(mfrow=c(2,3))

sresid <- resid(m4, type = "pearson")

hist(sresid)

fits <- fitted(m4)

plot(sresid ~ fits)

qqnorm(resid(m4))

qqline(resid(m4))


# Square root transformation

m1 <- lmer(sqrt(forage_days) ~ site*switched + thorax + longevity + mean_forage_time + guard + (1|colony),
data=foragers)

m2 <- lmer(sqrt(forage_days) ~ site*switched + thorax + longevity + mean_forage_time + (1|colony),
data=foragers)

AICc(m1,m2)

# keep m2


m3 <- lmer(sqrt(forage_days) ~ site*switched + thorax + longevity + (1|colony), data=foragers)

AICc(m2,m3)

# keep m2


m4 <- lmer(sqrt(forage_days) ~ site*switched + thorax + mean_forage_time + (1|colony), data=foragers)

AICc(m2,m4)

# keep m4
```

```
m5 <- lmer(sqrt(forage_days) ~ site*switched + mean_forage_time + (1|colony), data=foragers)

AICc(m4,m5)

# keep m4


m6 <- lmer(sqrt(forage_days) ~ switched + site + thorax + mean_forage_time + (1|colony), data=foragers)

AICc(m4,m6)

# keep m6


# Check assumptions

sresid <- resid(m6, type = "pearson")

hist(sresid)

fits <- fitted(m6)

plot(sresid ~ fits)

qqnorm(resid(m6))

qqline(resid(m6))


# Final Model

model24 <- lmer(sqrt(forage_days) ~ switched + site + thorax + mean_forage_time + (1|colony), data=foragers)

summary(model24)



## 25. Switched colony and Thorax

# Preliminary plots

ggplot(data=thorax,

	aes(x = switched, y = thorax)) +

	geom_boxplot()+

	stat_summary(fun = "mean",

	geom = "point",

	shape = 5, size = 3)
```

```
ggplot(data=thorax,

        aes(x = switched, y = thorax)) +

        geom_boxplot()+

        facet_grid(. ~ site)+

stat_summary(fun = "mean",

        geom = "point",

        shape = 5, size = 3)


# Model selection using AICc

m1 <- glmer(switched ~ thorax + longevity + mean_forage_time + forage_days + (1|colony), family = binomial
(link="cloglog"), data = thorax)

m2 <- glmer(switched ~ thorax + longevity + mean_forage_time + (1|colony), family = binomial
(link="cloglog"), data = thorax)

AICc(m1,m2)

# keep m2


m3 <- glmer(switched ~ thorax + longevity + (1|colony), family = binomial (link="cloglog"), data = thorax)

AICc(m2,m3)

# keep m3


# Check assumptions

devresid <- resid(model25, type="deviance")

hist(devresid)


# Final Model

model25 <- glmer(switched ~ thorax + longevity + (1|colony), family = binomial (link="cloglog"), data =
thorax)


### MAIN PLOTS ###

## 1. Guarding and Longevity

 bees_summary1 <- bees %>%
```

```
    group_by(guard) %>%
    summarise(mlongev = mean(longevity),
              sd_mlongev = sd(longevity),
              n_mlongev = n(),
              SE_mlongev = sd(longevity)/sqrt(n()))

p1 <- ggplot(bees_summary1, aes(guard, mlongev)) +
    geom_col(show.legend = FALSE, fill=c("skyblue1","steelblue4"),width=0.8)+
    geom_errorbar(aes(ymin = mlongev - sd_mlongev,
    ymax = mlongev + sd_mlongev), width=0.2)+
    theme_bw()+
    labs(x="",y="Mean Longevity (days)")+
    scale_x_discrete(labels=c("Not Guards", "Guards"))+
    scale_y_continuous(limits=c(0,30.3),expand = c(0, 0))+
    theme(axis.text=element_text(size=22),
    axis.title=element_text(size=22))

## 2. Guarding and No. foraging trips
bees_summary2 <- bees %>%
    group_by(guard) %>%
    summarise(mtrips = mean(forage_days),
              sd_mtrips = sd(forage_days),
              n_mtrips = n(),
              SE_mtrips = sd(forage_days)/sqrt(n()))

p2 <- ggplot(bees_summary2, aes(guard, mtrips, fill=c("steelblue1","tomato1"))) +
    geom_col(show.legend = FALSE, fill=c("skyblue1","steelblue4"),width=0.8)+
    geom_errorbar(aes(ymin = mtrips - sd_mtrips,
    ymax = mtrips + sd_mtrips), width=0.2)+
    theme_bw()+
```

```
	labs(x="",y="Mean No. Foraging Trips per Day")+
	scale_x_discrete(labels=c("Not Guards", "Guards"))+
	scale_y_continuous(limits=c(0,15.3),expand = c(0, 0))+
	theme(axis.text=element_text(size=22),
	axis.title=element_text(size=22))

plot_grid(p1, p2, labels=c("a)", "b)"),label_size = 20, hjust = -0.3, ncol = 2, nrow = 1)

## 3. Gipps stealing and Mean foraging
bees_summary3 <- foragersG %>%
	group_by(stole) %>%
	summarise(mforage = mean(mean_forage_time),
			sd_mforage = sd(mean_forage_time),
			n_mforage = n(),
			SE_mforage = sd(mean_forage_time)/sqrt(n()))

p3 <- ggplot(bees_summary3, aes(stole, mforage, fill=c("steelblue1","tomato1"))) +
	geom_col(show.legend = FALSE, fill=c("skyblue1","steelblue4"),width=0.8)+
	geom_errorbar(aes(ymin = mforage - sd_mforage,
	ymax = mforage + sd_mforage), width=0.2)+
	theme_bw()+
	labs(x="",y="Mean Length of Foraging Trip (hrs)")+
	scale_x_discrete(labels=c("Not Stealing", "Stealing"))+
	scale_y_continuous(limits=c(0,2.04),expand = c(0, 0))+
	theme(axis.text=element_text(size=22),
	axis.title=element_text(size=22))

## 4. Gipps stealing and No. foraging trips
bees_summary4 <- beesG %>%
	group_by(stole) %>%
```

```
        summarise(mtrips = mean(forage_days),
                        sd_mtrips = sd(forage_days),
                        n_mtrips = n(),
                        SE_mtrips = sd(forage_days)/sqrt(n()))

p4 <- ggplot(bees_summary4, aes(stole, mtrips, fill=c("steelblue1","tomato1"))) +
        geom_col(show.legend = FALSE, fill=c("skyblue1","steelblue4"),width=0.8)+
        geom_errorbar(aes(ymin = mtrips - sd_mtrips,
        ymax = mtrips + sd_mtrips), width=0.2)+
        theme_bw()+
        labs(x="",y="Mean No. Foraging Trips per Day")+
        scale_x_discrete(labels=c("Not Stealing", "Stealing"))+
        scale_y_continuous(limits=c(0,15.3),expand = c(0, 0))+
        theme(axis.text=element_text(size=22),
        axis.title=element_text(size=22))

plot_grid(p3, p4, labels=c("a)", "b)"),label_size = 20, hjust = -0.3, ncol = 2, nrow = 1)

### APPENDIX PLOTS ###
## 1. Guarding and Thorax
 bees_summary5 <- thorax %>%
        group_by(guard) %>%
        summarise(mthorax = mean(thorax),
                        sd_mthorax = sd(thorax),
                        n_mthorax = n(),
                        SE_mthorax = sd(thorax)/sqrt(n()))

p5 <- ggplot(bees_summary5, aes(guard, mthorax)) +
        geom_col(show.legend = FALSE, fill=c("skyblue1","steelblue4"),width=0.8)+
        geom_errorbar(aes(ymin = mthorax - sd_mthorax,
```

```
        ymax = mthorax + sd_mthorax), width=0.2)+
        theme_bw()+
        labs(x="",y="Mean Thorax Width (mm)")+
        scale_x_discrete(labels=c("Not Guards", "Guards"))+
        scale_y_continuous(limits=c(0,8.1),expand = c(0, 0))+
        theme(axis.text=element_text(size=22),
        axis.title=element_text(size=22))

## 2. Guarding and Mean Forgaging time
bees_summary6 <- foragers %>%
        group_by(guard) %>%
        summarise(mforage = mean(mean_forage_time),
                        sd_mforage = sd(mean_forage_time),
                        n_mforage = n(),
                        SE_mforage = sd(mean_forage_time)/sqrt(n()))

p6 <- ggplot(bees_summary6, aes(guard, mforage, fill=c("steelblue1","tomato1"))) +
        geom_col(show.legend = FALSE, fill=c("skyblue1","steelblue4"),width=0.8)+
        geom_errorbar(aes(ymin = mforage - sd_mforage,
        ymax = mforage + sd_mforage), width=0.2)+
        theme_bw()+
        labs(x="",y="Mean Length of Foraging Trip (hrs)")+
        scale_x_discrete(labels=c("Not Guards", "Guards"))+
        scale_y_continuous(limits=c(0,2.04),expand = c(0, 0))+
        theme(axis.text=element_text(size=22),
        axis.title=element_text(size=22))

plot_grid(p5, p6, labels=c("a)", "b)"),label_size = 20, hjust = -0.3, ncol = 2, nrow = 1)

## 3. Gipps Stealing and Thorax
```

```
bees_summary7 <- thoraxG %>%
        group_by(stole) %>%
        summarise(mthorax = mean(thorax),
                        sd_mthorax = sd(thorax),
                        n_mthorax = n(),
                        SE_mthorax = sd(thorax)/sqrt(n()))

p7 <- ggplot(bees_summary7, aes(stole, mthorax)) +
        geom_col(show.legend = FALSE, fill=c("skyblue1","steelblue4"),width=0.8)+
        geom_errorbar(aes(ymin = mthorax - sd_mthorax,
        ymax = mthorax + sd_mthorax), width=0.2)+
        theme_bw()+
        labs(x="",y="Mean Thorax Width (mm)")+
        scale_x_discrete(labels=c("Not Stealing", "Stealing"))+
        scale_y_continuous(limits=c(0,8.1),expand = c(0, 0))+
        theme(axis.text=element_text(size=22),
        axis.title=element_text(size=22))

## 4. Gipps Stealing and Longevity
 bees_summary8 <- beesG %>%
        group_by(stole) %>%
        summarise(mlongev = mean(longevity),
                        sd_mlongev = sd(longevity),
                        n_mlongev = n(),
                        SE_mlongev = sd(longevity)/sqrt(n()))

p8 <- ggplot(bees_summary8, aes(stole, mlongev)) +
        geom_col(show.legend = FALSE, fill=c("skyblue1","steelblue4"),width=0.8)+
        geom_errorbar(aes(ymin = mlongev - sd_mlongev,
        ymax = mlongev + sd_mlongev), width=0.2)+
```

```
	theme_bw()+
	labs(x="",y="Mean Longevity (days)")+
	scale_x_discrete(labels=c("Not Stealing", "Stealing"))+
	scale_y_continuous(limits=c(0,30.3),expand = c(0, 0))+
	theme(axis.text=element_text(size=22),
	axis.title=element_text(size=22))

plot_grid(p7, p8, labels=c("a)", "b)"),label_size = 20, hjust = -0.3, ncol = 2, nrow = 1)

## 5. Switching and Thorax
bees_summary9 <- thorax %>%
	group_by(switched) %>%
	summarise(mthorax = mean(thorax),
			sd_mthorax = sd(thorax),
			n_mthorax = n(),
			SE_mthorax = sd(thorax)/sqrt(n()))

p9 <- ggplot(bees_summary9, aes(switched, mthorax)) +
	geom_col(show.legend = FALSE, fill=c("skyblue1","steelblue4"),width=0.8)+
	geom_errorbar(aes(ymin = mthorax - sd_mthorax,
	ymax = mthorax + sd_mthorax), width=0.2)+
	theme_bw()+
	labs(x="",y="Mean Thorax Width (mm)")+
	scale_x_discrete(labels=c("Stayed", "Switched"))+
	scale_y_continuous(limits=c(0,8.1),expand = c(0, 0))+
	theme(axis.text=element_text(size=15.4),
	axis.title=element_text(size=15.4))

## 6. Switching and Longevity
bees_summary10 <- bees %>%
```

```
    group_by(switched) %>%
    summarise(mlongev = mean(longevity),
              sd_mlongev = sd(longevity),
              n_mlongev = n(),
              SE_mlongev = sd(longevity)/sqrt(n()))

p10 <- ggplot(bees_summary10, aes(switched, mlongev)) +
    geom_col(show.legend = FALSE, fill=c("skyblue1","steelblue4"),width=0.8)+
    geom_errorbar(aes(ymin = mlongev - sd_mlongev,
    ymax = mlongev + sd_mlongev), width=0.2)+
    theme_bw()+
    labs(x="",y="Mean Longevity (days)")+
    scale_x_discrete(labels=c("Stayed", "Switched"))+
    scale_y_continuous(limits=c(0,25.3),expand = c(0, 0))+
    theme(axis.text=element_text(size=15.4),
    axis.title=element_text(size=15.4))


## 7. Switching and Mean foraging time
bees_summary11 <- foragers %>%
    group_by(switched) %>%
    summarise(mforage = mean(mean_forage_time),
              sd_mforage = sd(mean_forage_time),
              n_mforage = n(),
              SE_mforage = sd(mean_forage_time)/sqrt(n()))

p11 <- ggplot(bees_summary11, aes(switched, mforage, fill=c("steelblue1","tomato1"))) +
    geom_col(show.legend = FALSE, fill=c("skyblue1","steelblue4"),width=0.8)+
    geom_errorbar(aes(ymin = mforage - sd_mforage,
    ymax = mforage + sd_mforage), width=0.2)+
```

```
	theme_bw()+
	labs(x="",y="Mean Length of Foraging
Trip (hrs)")+
	scale_x_discrete(labels=c("Stayed", "Switched"))+
	scale_y_continuous(limits=c(0,2.04),expand = c(0, 0))+
	theme(axis.text=element_text(size=15.4),
	axis.title=element_text(size=15.4))

## 8. Switching and No. foraging trips
 bees_summary12 <- bees %>%
	group_by(switched) %>%
	summarise(mtrips = mean(forage_days),
			sd_mtrips = sd(forage_days),
			n_mtrips = n(),
			SE_mtrips = sd(forage_days)/sqrt(n()))

p12 <- ggplot(bees_summary12, aes(switched, mtrips, fill=c("steelblue1","tomato1"))) +
	geom_col(show.legend = FALSE, fill=c("skyblue1","steelblue4"),width=0.8)+
	geom_errorbar(aes(ymin = mtrips - sd_mtrips,
	ymax = mtrips + sd_mtrips), width=0.2)+
	theme_bw()+
	labs(x="",y="Mean No. Foraging Trips
per Day")+
	scale_x_discrete(labels=c("Stayed", "Switched"))+
	scale_y_continuous(limits=c(0,15.3),expand = c(0, 0))+
	theme(axis.text=element_text(size=15.4),
	axis.title=element_text(size=15.4))

plot_grid(p9, p10, p11, p12, labels=c("a)", "b)","c)","d)"),label_size = 14, vjust = 0.9,hjust = -0.3, ncol = 2, nrow = 2)
```
